# A general model for analysis of linear and hyperbolic enzyme inhibition mechanisms

**DOI:** 10.1002/2211-5463.70128

**Published:** 2025-09-24

**Authors:** Rafael S. Chagas, Sandro R. Marana

**Affiliations:** ^1^ Departamento de Bioquímica, Instituto de Química Universidade de São Paulo Brazil

**Keywords:** GH1 β‐glucosidases, imidazole, inhibition mechanisms, Tris

## Abstract

The mechanisms of reversible inhibitors with a single binding site on enzymes are usually divided into two basic groups: linear and hyperbolic (or partial). Each of these two groups is subdivided into three types: competitive, non‐competitive and mixed. These six mechanisms are often considered separate identities. Here, prompted by the characterization of the inhibition of the wild‐type and mutant β‐glucosidase Sfβgly by imidazole and 2‐amino‐2‐(hydroxymethyl)‐1,3‐propanediol (i.e. Tris), we developed a unifying enzyme kinetic model that integrates these six basic inhibition mechanisms into one. From this model, we deduced a general enzyme kinetic equation that, through modulation of simple parameters (i.e. the relative inhibitor affinity for two binding sites and the reactivity of the enzyme–substrate–inhibitor complex) is converted into the particular kinetic equation of each of those six inhibition mechanisms. In short, we conclude that the six fundamental inhibition mechanisms, linear and hyperbolic, are not separate behaviors but facets of the same general kinetic model presented here.

## Graphical abstract

We developed a general enzyme kinetic model that integrates these six basic inhibition mechanism onto a single one. From this model, we deduced a general enzyme kinetic equation that through modulation of simple parameters, γ (the relative inhibitor affinity for two binding sites) and β (the reactivity of the enzyme–substrate–inhibitor complex), is converted into the particular kinetic equation of each of those six inhibition mechanism.

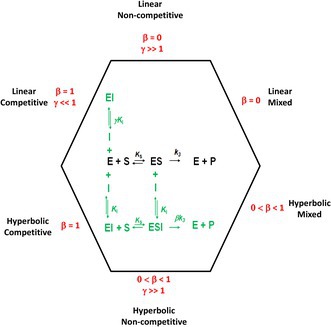

**Abbreviations**
NPβglc: *p*‐nitrophenyl β‐glucoside
PDB: Protein Data Bank
Sfβgly: GH1 β‐glucosidase from *Spodoptera frugiperda* (PDB: 5CG0)
Tris: 2‐amino‐2‐(hydroxymethyl)‐1,3‐propanediol

## Enzyme inhibition mechanism: a brief overview

The mechanisms of the reversible inhibitors that have only a single binding site on enzymes are usually divided into two groups: linear and hyperbolic or partial. Both groups are called simple mechanisms as a result of the single binding site. Assuming initial rate (*v*_0_) conditions, they are delimited based on the behavior of the slope (*K*_s_/*k*_cat app_) and intercept (1/*k*_cat app_) lines in the Lineweaver–Burk plot (1/*v*_0_ × 1/[*S*]), which were determined under different inhibitor concentrations. So, as their denomination indicates, simple linear inhibitors render linear correlations when slope or intercept are plotted *versus* [I]. In agreement, simple hyperbolic (partial) inhibitors generate curved lines (hyperboles) in that plot, where the slope or intercept levels off at high [I].

These two groups can be further divided into types: competitive, non‐competitive and mixed, which are identified by the relative positioning of the lines in the Lineweaver–Burk plots obtained with several [I]. Basically, these inhibition types differ in the positioning of the point where the different lines cross. Hence, regardless of the group, linear or hyperbolic, they are called an intercepting mechanism.

Conversely, uncompetitive inhibitors do not belong to the intercepting group because they produce parallel lines in the Lineweaver–Burk plots.

In short, considering only the intercepting cases, there are six basic simple inhibition mechanisms, which are commonly represented in textbooks [1], but, for clarity purposes, they are also shown in the Fig. S1. Finally, as gathered from the classificatory effort, the inhibition mechanisms are understood as separate identities.

Formerly, in a context of few structural data available, the inhibition mechanism studies provided a path to idealize schematic models of the enzyme structures. Currently, in a richer structural data environment, these inhibition models can be used to deposit additional layers of functional information on the enzyme structure [2, 3, 4, 5, 6]. However, because these inhibition mechanistic models were not developed to represent microscopic molecular steps, when they are combined with structural data, sometimes tensions arise. Nevertheless, instead of representing a limitation, these frictions are opportunities to develop models amenable to smoothly integrating inhibition and structural binding data, harnessing the ease of the initial rate experiments to improve the functional characterization of enzymes.

## GH1 β‐glucosidase sfβgly as a working model for the development of a new enzyme inhibition model

**Fig. 1:**
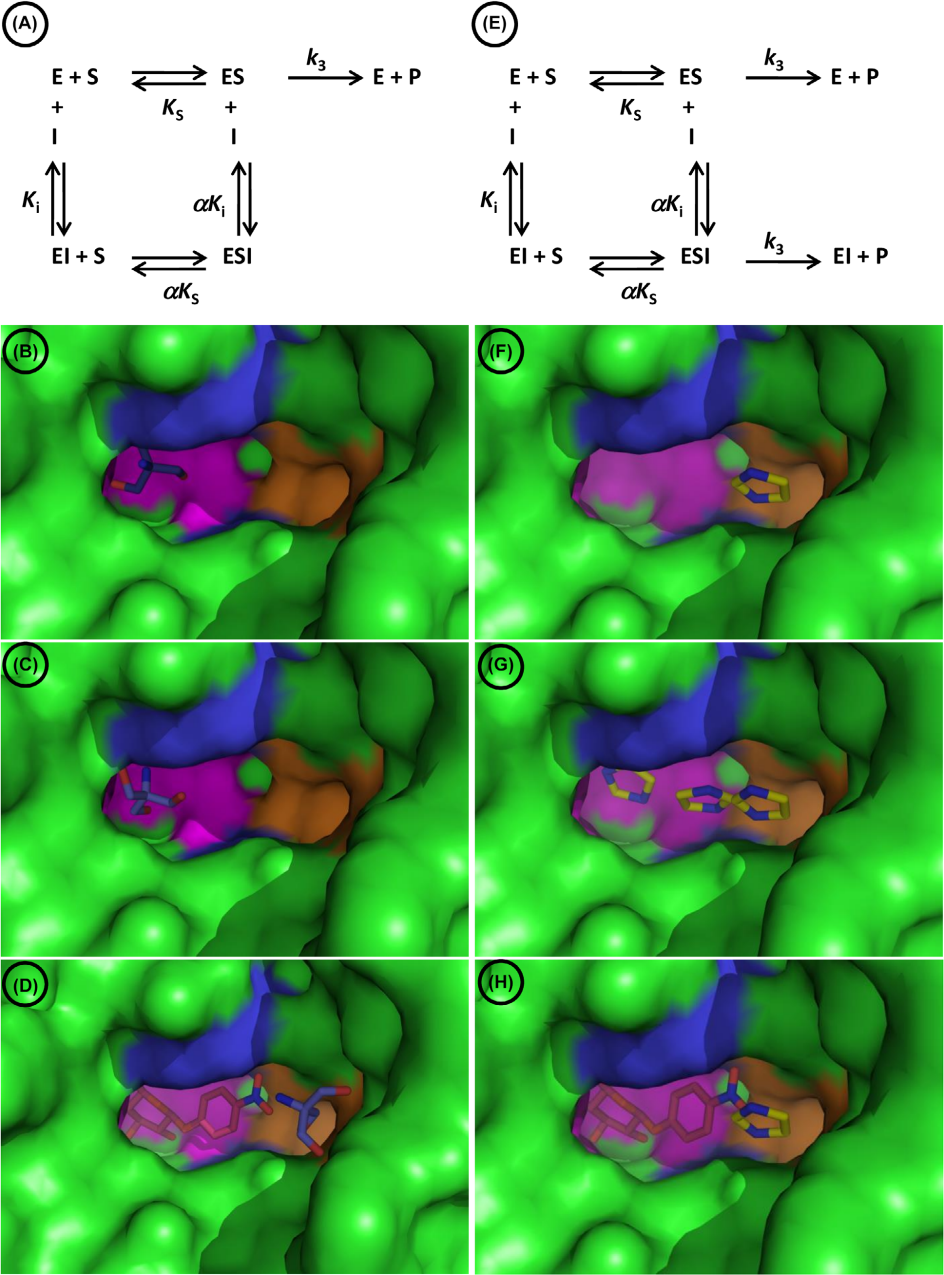
Mechanistic and structural description of the inhibition of the Sfβgly by Tris and imidazole. Structures show a close‐up of the Sfβgly active site (PDB 5CG0). The subsite −1 is colored in pink, the subsite +1 is in blue and a lateral pocket in the active site opening is marked in orange. (A) Tris inhibition mechanism [9], which is classified as intercepting, linear and mixed [1]. (B) Tris docked within the subsite −1 of the Sfβgly active site. Based on Chagas and Marana [9]. (C) Crystallographic structure of Tris bound within the Sfβgly active site (PDB: 5CG0). Based on Tamaki *et al*. [7]. (D) Putative ESI complex generated by docking of Tris (dark blue and red) in the Sfβgly structure in which the active site was previously occupied by NPβglc (*p*‐nitrophenyl β‐glucoside) (red). Tris is in the region of the lateral pocket in the active site opening (orange) (E) Imidazole inhibition mechanism [8], which is classified as intercepting, hyperbolic and partial competitive. (F) Computational docking showing the Imidazole bound in a lateral pocket in the active site opening. Based on Chagas *et al*. [8]. (G) Simultaneous representation of three different docking solutions of imidazole in the Sfβgly: active site subsites −1 and +1, and the lateral pocket in the active site opening. Based on Chagas *et al*. [8]. (H) Putative ESI complex involving NPβglc (red), within the Sfβgly active site (subsites −1 and +1, pink and blue, respectively), and imidazole (yellow and blue), in the lateral pocket in the active site opening (orange).

Having that goal in mind, our working model is the previously reported reversible inhibition of the GH1 β‐glucosidase Sfβgly [Protein Data Bank (PDB): 5CG0) [7] by imidazole and 2‐amino‐2‐(hydroxymethyl)‐1,3‐propanediol (i.e. Tris) [8, 9]. Sfβgly, a digestive GH1 β‐glucosidase from the fall armyworm *Spodoptera frugiperda*, has a tunnel‐shaped active site, where short oligocellosaccharides (polymerization degree from 2 to 4), non‐branched alkyl β‐glucosides (4–12 carbons) and bulky aryl β‐glucosides can fit [10]. The monosaccharide at the non‐reducing extremity of the substrate (glycone) binds in the dead end of the tunnel (subsite −1), whereas the group (saccharide or not) linked to the glycone via the β‐glycosidic bond is positioned towards the active site opening, a region that comprises the subsite +1 (Fig. 1).

Imidazole acts as a simple hyperbolic competitive inhibitor, also known as partial competitive, whereas Tris functions as a simple linear mixed type (Fig. 1) [8, 9]. Despite their clear particularities and distinct identities, there is indeed some common ground between these mechanisms. For example, both mechanisms present a ternary complex ESI, indicating that the inhibitor (either imidazole or Tris) has a single binding site, different from that of the substrate, which is found in both the free enzyme (E) and the enzyme–substrate complex (ES) (Fig. 1). Thus, this same single site is occupied by a single inhibitor molecule in the EI and ESI complexes. The same inhibition mechanism was observed when using synthetic and natural substrates, as *p*‐nitrophenyl β‐glucoside (NPβglc) and cellobiose, respectively [8, 9].

In agreement, computational docking suggested that a side pocket in the Sfβgly active site opening could be the imidazole binding site, leaving the −1 and +1 subsites available for the substrate (Fig. 1). Hence, such a lateral binding site would be compatible with the formation of the ESI complex, which, as noted above, is a requirement for the partial competitive inhibition mechanism observed experimentally [1, 8] (Fig. 1). The crater‐shaped opening of the Sfβgly active site, adapted to bind bulky aryl groups as seen in the substrates amygdalin and methylumbelliferyl β‐glucoside [11], is large enough to comprise that pocket and to enable the simultaneous interaction with the substrate (NPβglc) and the inhibitor (imidazole), as proposed in the putative ESI complex (Fig. 1). Even so, computational docking data also pointed out that imidazole could potentially bind at several sites along the Sfβgly active site, from the −1 subsite, at the dead end of the active site, up to the +1 subsite, close to its wide opening [8] (Fig. 1). Such a prediction does not fit into the inhibition mechanism observed experimentally.

This imperfect match between structural and inhibition data shows again for Tris. It binds within the −1 subsite of the Sfβgly crystallographic structure [7], an interaction also detected by computational docking. However, such interaction is typical of competitive inhibition, which is not observed experimentally for Tris and Sfβgly. On the other side, Tris can also bind in a lateral pocket of the active site opening when docked into the ES complex (NPβglc–Sfβgly complex) (Fig. 1). This interaction is consistent with the detection of a linear mixed inhibition mechanism, which includes the formation of an unproductive ESI complex.

In short, a representation combining these observations results in some tension. The interactions of imidazole and Tris around the Sfβgly active site opening are compatible with their experimental inhibition mechanisms, which include the formation of ESI complexes (Fig. 1). Nonetheless, as a consequence, this consideration would imply that the observed interactions of imidazole and Tris in the internal portion of the active site (−1 and +1 subsites) should be irrelevant, because they do not influence the inhibition mechanism.

This apparent contradiction could be solved if imidazole and Tris could really interact with multiple spots along the active site, as shown by their computational dockings (Fig. 1). However, this hypothetical binding at multiple sites is apparently incompatible with their experimental inhibition mechanisms, which are based on the interaction of each inhibitor at a single site (Fig. 1).

To develop a model to dissipate such tension, we followed an experimental and theoretical approach.

First, aiming to confirm the presence of the inhibitor binding site in the side pocket of the active site opening, single mutations were introduced at Sfβgly residues S247, N249 and F251, which interact with imidazole in that pocket. Residue S247 was replaced by Y, N249 was exchanged for Q and A, and F251 was replaced by A. The inhibitory effect of imidazole and Tris on the mutant Sfβgly was characterized and compared to the wild‐type enzyme.

Finally, to interpret and integrate the experimental observations gathered with the wild‐type and mutant Sfβgly, we developed a general inhibition model that unifies six fundamental inhibition mechanisms (linear and hyperbolic) previously described in the literature. It is noteworthy that this model is really general in the sense that it is not specific for the inhibitory effect of imidazole and Tris on Sfβgly, but it is an effective tool for characterizing the mechanisms of a broad range of small molecule inhibitors presenting single or multiple binding sites in enzymes.

## Material and methods

### Site‐directed mutagenesis experiments

Site‐directed mutagenesis experiments were performed using QuikChange Lightning Site‐Directed Mutagenesis Kit (Agilent Technologies, Santa Clara, CA, USA) in accordance with the manufacturer's instructions. A pET46 EK LIC vector (Novagen, Darmstadt, Germany) coding for the wild‐type Sfβgly was used as a template. Such vector was already available in the laboratory [12]. Mutagenic primers were designed following the guidelines of the QuikChange Lightning Site‐Directed Mutagenesis kit. Mutations were confirmed by DNA sequencing.

### Expression and purification of the mutant Sfβgly

The Sfβgly mutants were produced and purified as previously reported [8]. Briefly, the pET46 vector coding for the mutant Sfβgly was introduced into BL21(DE3) bacteria. Transformed cells were cultivated in liquid LB medium (200 ***g*** at 37 °C) and induced with 1.0 mm isopropyl β‐thiogalactopyranoside (200 ***g*** at 25 °C for 16 h) to produce the recombinant proteins [3]. Following that, the induced bacteria were ruptured by using sonication. The lysate was centrifuged (4000 ***g*** at 4 °C for 30 min) and employed for the mutant Sfβgly purification by using Ni‐NTA agarose resin (Qiagen, Hilden, Germany). Considering that the purified mutant Sfβgly samples were eluted in high imidazole concentration (0.5 m), they were subjected to buffer exchange using PD Minitrap G‐25 columns (Cytiva, Marlborough, MA, USA) previous to any additional experiment. Hence, the final enzyme samples were stored in 100 mm phosphate buffer, pH 6, at 4 °C. Protein homogeneity was assessed by SDS/PAGE [13] and protein concentration was measured using the bicinchoninic acid assay [14].

### Enzyme inhibition assays

#### Inhibition of mutant Sfβgly by imidazole and tris

The initial rate of hydrolysis (*v*_0_) of at least 10 different concentrations of *p*‐nitrophenyl β‐glucoside (NPβglc), ranging from 0.625 to 25 mm, was determined in the presence of five different imidazole concentrations (0–120 mm) or five concentrations of Tris (0–120 mm). Initial rates were determined from three separate reactions with a single enzyme sample performed at 30 °C. NPβglc and imidazole were prepared in 100 mm phosphate buffer, pH 6.0. The product, *p*‐nitrophenolate, was detected at 415 nm after the addition of 0.5 m Na_2_CO_3_ to the reaction mix. NaCl was added to adjust the ionic strength among reactions, with 120 mm imidazole as the reference. Data were analyzed using Lineweaver–Burk plots. The complete 1/*v*_0_ *versus* 1/[S] data set is available as Data S1. Linear fits were accepted when showing *R* ^2^ values higher than 0.9. The *K*_i_ and α factor were determined using procedures appropriate for the inhibition mechanism [1] and are expressed as the mean ± SD.

#### Molecular docking of imidazole and tris in the Sfβgly

The crystallographic structures of the Sfβgly (PDB: 5CG0) [2] *Neotermes koshunesis* β‐glucosidase in complex with NPβglc (PDB: 3AI0) [15] were structurally superposed using pymol (The PyMOL Molecular Graphics System, version 0.99; Schrödinger, LLC, New York, NY, USA). Then the merged coordinates of Sfβgly and NPβglc were used in the molecular docking performed in autodock vina as previously described [8, 9, 16, 17]. The coordinates for imidazole and Tris were generated using gabedit, version 2.5.1 [18], and both were docked in their mono‐protonated states (+1 charge). The grid box coordinates covered all protein atoms. The exhaustiveness was set to 10. The nine best models were visually inspected in pymol.

## Results and Discussion

As mentioned above, the combination of the structural and mechanistic data of the Sfβgly inhibition produces some tension, particularly regarding the number of the inhibitor binding sites. Two, not excluding, options are placed: a side pocket in the active site opening and the active site *per se*. Aiming to obtain more data about the inhibitor binding sites and how they eventually determine the inhibition mechanism, four mutant Sfβgly (S247Y, N249A, N249Q and F251A), containing single replacements at the side pocket of the active site, were produced as recombinant proteins in *Escherichia coli* and purified following the procedures previously published (Fig. S2) [8]. These enzymes were used to determine the initial rate of hydrolysis of different NPβglc concentrations in the presence of either imidazole or Tris, each tested separately at several concentrations. The data were analyzed using the Lineweaver–Burk plot to determine the inhibition mechanism, a classical enzyme kinetics approach [1].

Next, the effects of each mutation on the inhibitors will be presented separately. The findings will then be combined and discussed.

### Mutational effects on imidazole inhibition

The mutations in the residues that form the lateral pocket at the active site opening produced diverse effects on imidazole inhibition mechanisms.

**Fig. 2:**
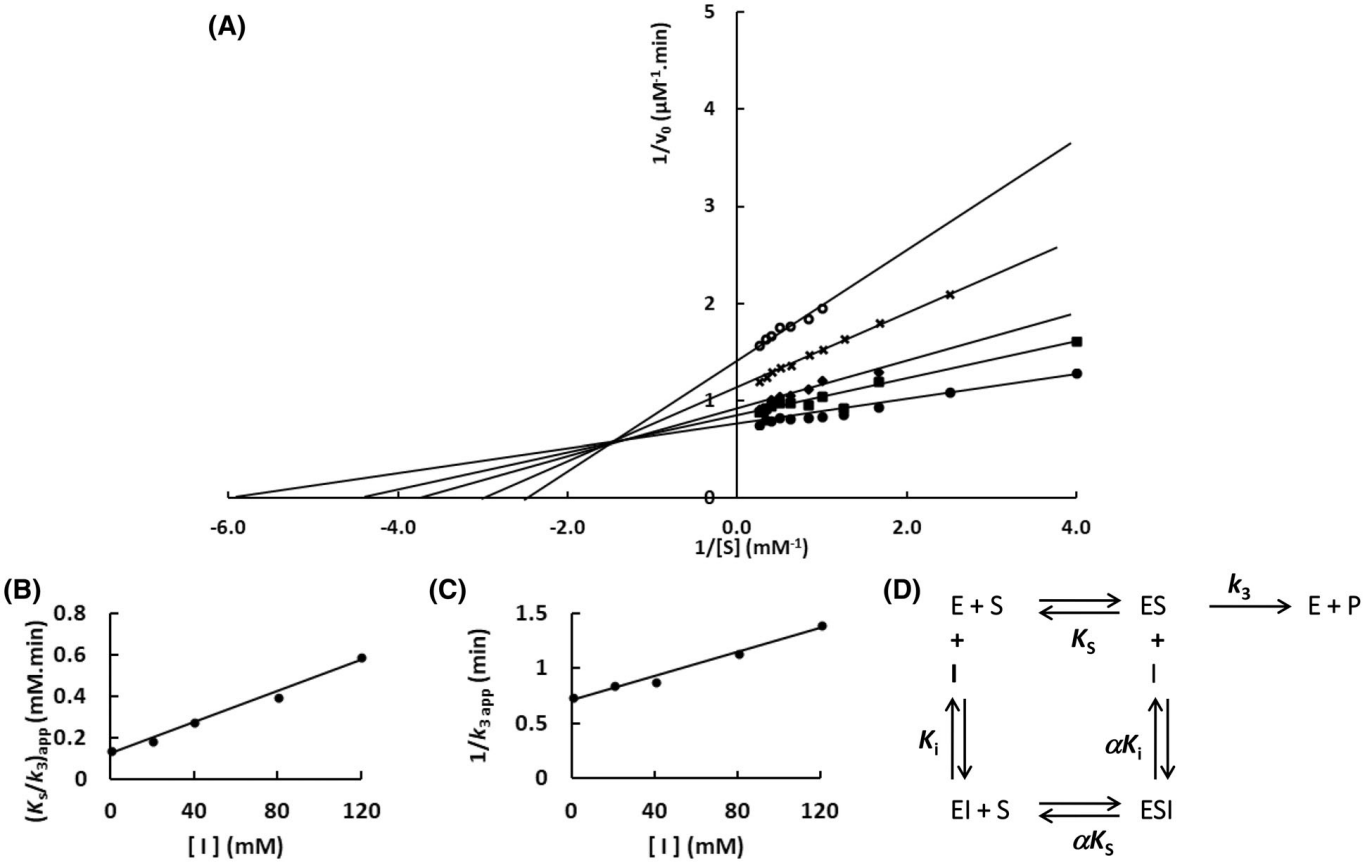
Determination of the Imidazole inhibition mechanism of the mutant Sfβgly S247Y. (A) Effect of different Imidazole concentrations (●, 0; ■, 20 mm; ◆, 40 mm; **x**, 80 mm; ○, 120 mm) on the initial rate of hydrolysis of the substrate NPβglc shown in Lineweaver–Burk plots. (B) Effect of imidazole concentration on the apparent *K*_s_/*k*_3_ (calculated from the line slope). (C) Effect of imidazole concentration on the apparent 1/*k*_3_ (calculated from the line intercept). Rates are the mean of three determinations of the product formed using the same enzyme sample. (D) Linear mixed inhibition mechanism (intersecting, linear, mixed). S, substrate NPβglc; I, inhibitor imidazole; E, enzyme Sfβgly; P, product; *K*_s_, dissociation constant of the ES complex; *K*_i_, dissociation constant of the EI complex; α*K*_i_, dissociation constant of the ESI complex; α, factor that represents the mutual hindering effect between S and I (α > 1); *k*_3_, rate constant of product formation [1].

**Fig. 3:**
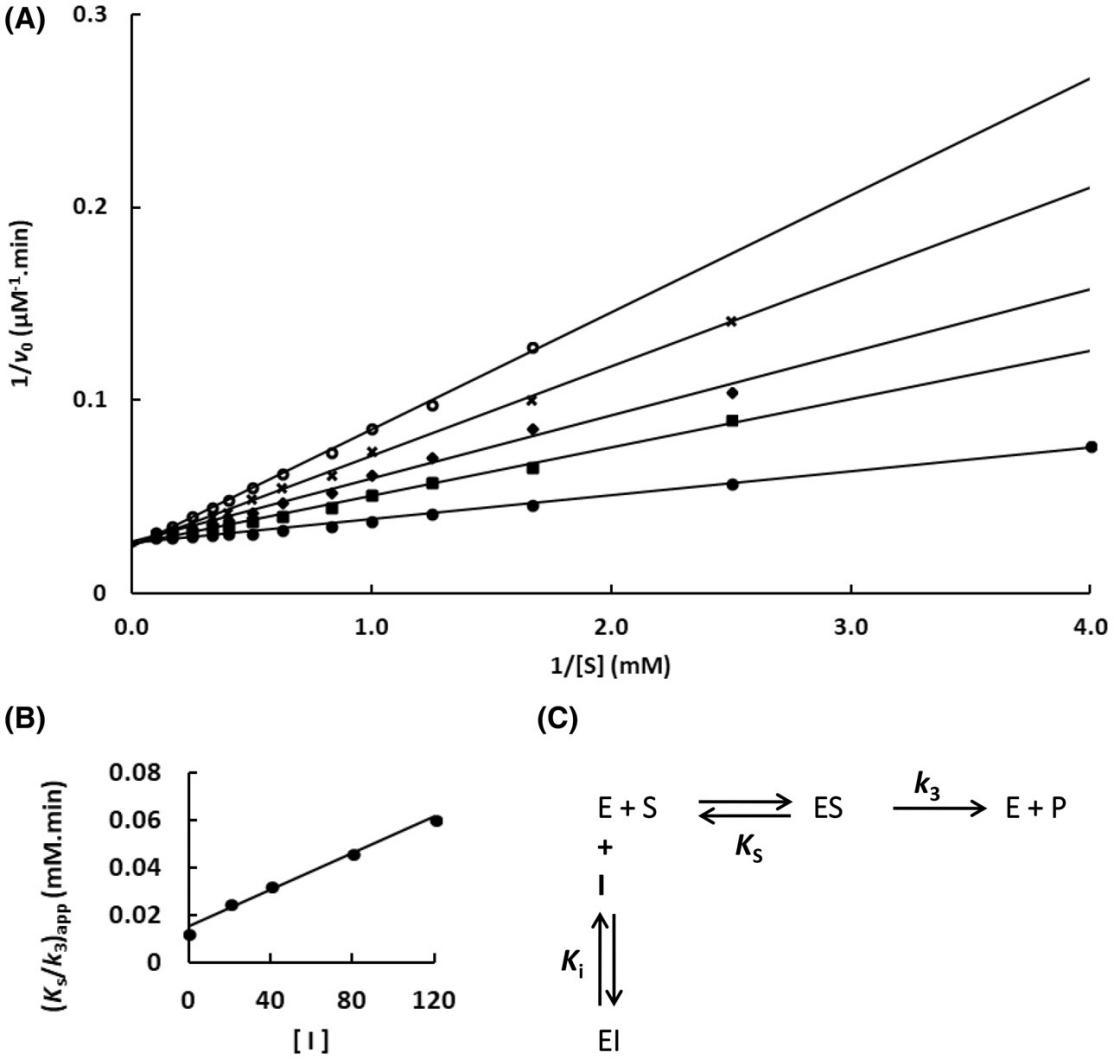
Determination of the imidazole inhibition mechanism in the mutant Sfβgly N249Q. (A) Effect of different Imidazole concentrations (●, 0; ■, 20 mm; ◆, 40 mm; **x**, 80 mm; ○, 120 mm) on the initial rate of hydrolysis of the substrate NPβglc shown in Lineweaver–Burk plots. (B) Effect of the Imidazole concentration on the apparent *K*_s_/*k*_3_ (calculated from the line slope). Rates are the mean of three determinations of the product formed using the same enzyme sample. (C) Linear competitive inhibition mechanism (intersecting, linear, competitive). S, substrate NPβglc; I, inhibitor imidazole; E, enzyme Sfβgly; P, product; *K*_s_, dissociation constant of the ES complex; *K*_i_, dissociation constant of the EI complex; *k*_3_, rate constant of product formation [1].

**Fig. 4:**
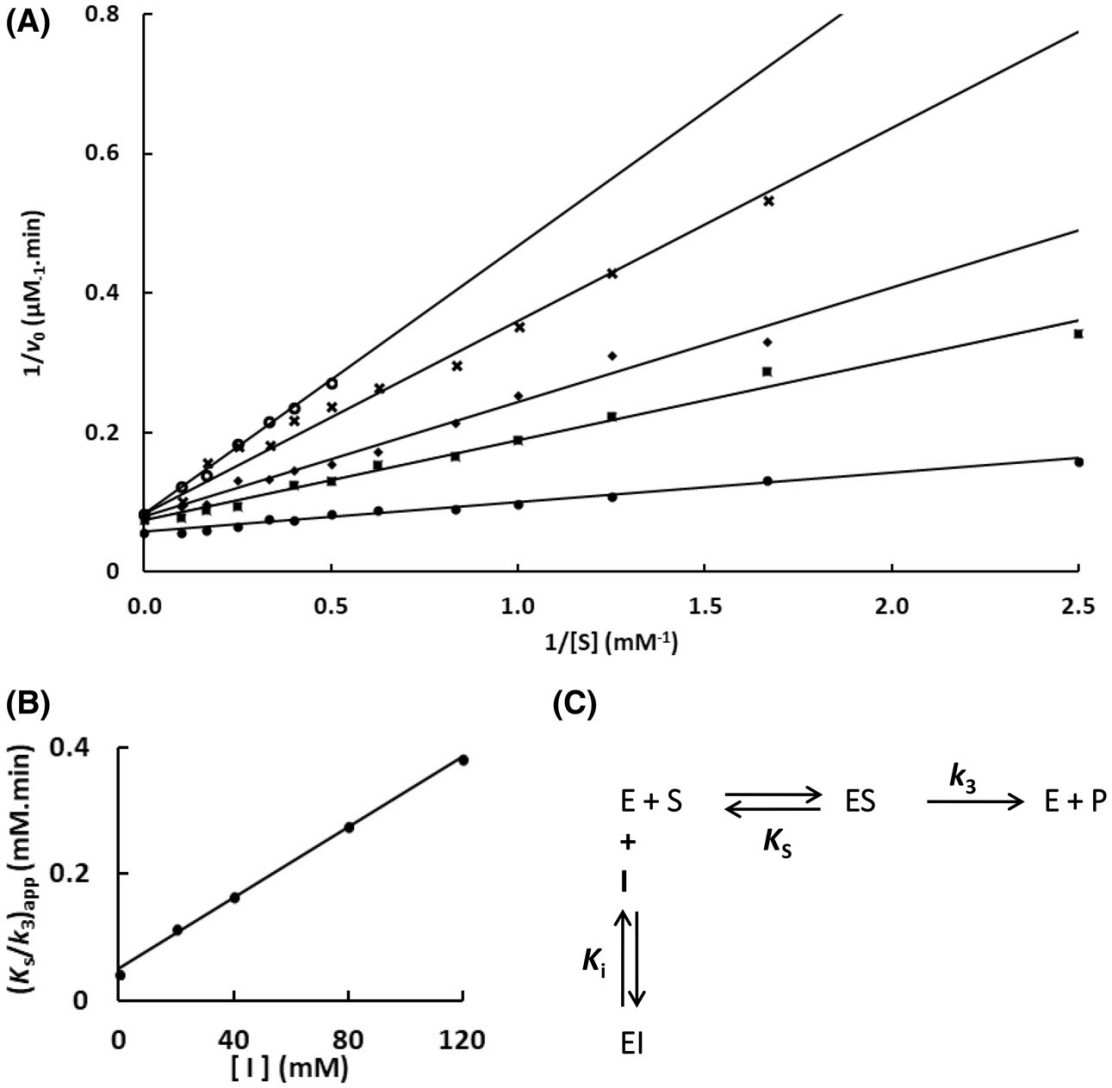
Determination of the Imidazole inhibition mechanism in the mutant Sfβgly N249A. (A) Effect of different Imidazole concentrations (●, 0; ■, 20 mm; ◆, 40 mm; **x**, 80 mm; ○, 120 mm) on the initial rate of hydrolysis of the substrate NPβglc shown in Lineweaver–Burk plots. (B) Effect of the imidazole concentration on the apparent *K*_s_/*k*_3_ (calculated from the line slope). Rates are the mean of three product determinations using the same enzyme sample. (C) Linear competitive inhibition mechanism (intersecting, linear, competitive). S, substrate NPβglc; I, inhibitor imidazole; E, enzyme Sfβgly; P, product; *K*_s_, dissociation constant of the ES complex; *K*_i_, dissociation constant of the EI complex; *k*_3_, rate constant of product formation [1].

**Fig. 5:**
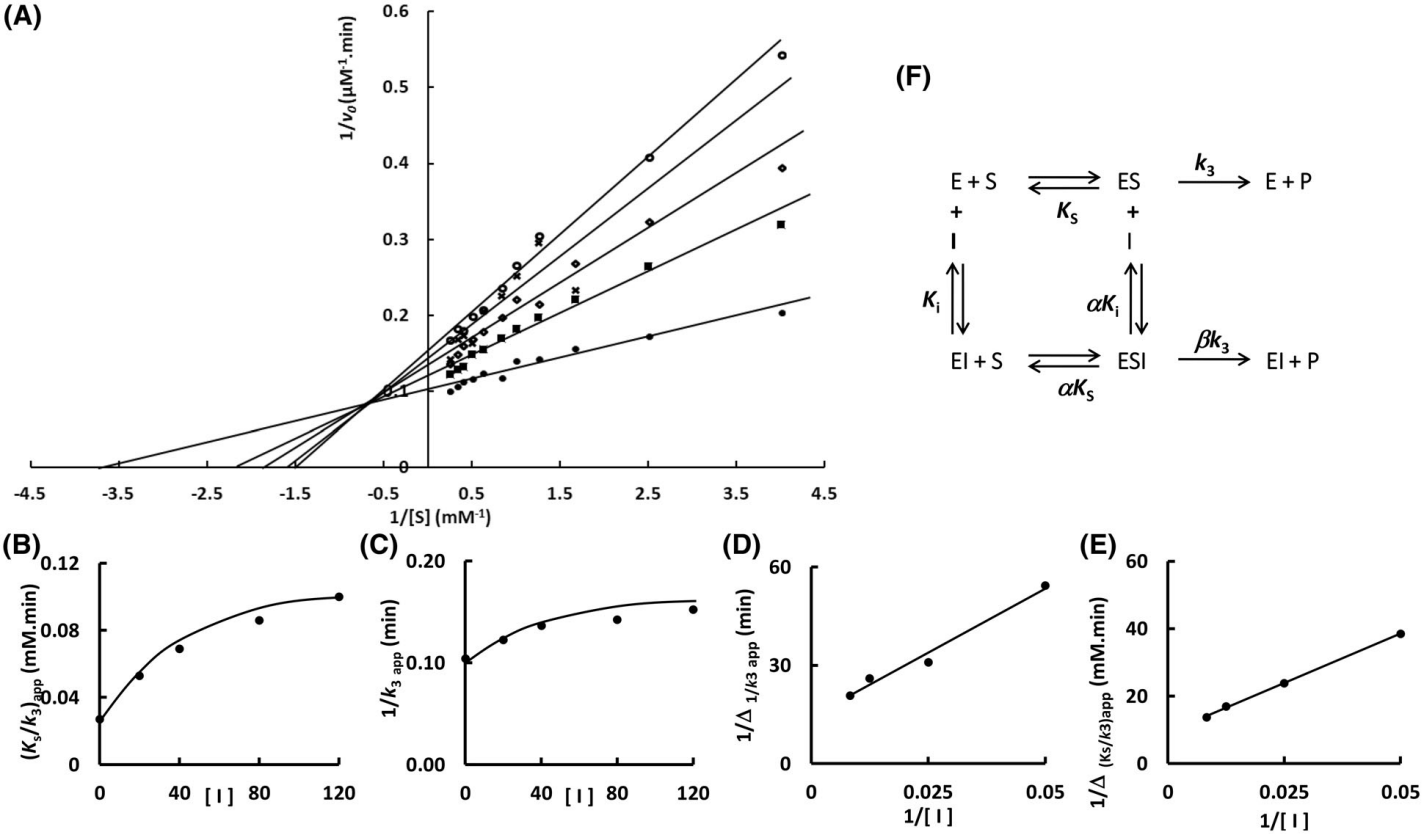
Determination of the Imidazole inhibition mechanism in the mutant Sfβgly F251A. (A) Effect of different Imidazole concentrations (●, 0; ■, 20 mm; ◆, 40 mm; **x**, 80 mm; ○, 120 mm) on the initial rate of hydrolysis of the substrate NPβglc shown in Lineweaver–Burk plots. (B) Effect of the Imidazole concentration on the apparent *K*_s_/*k*_3_ (calculated from the line slope). (C) Effect of the Imidazole concentration on the apparent 1/*k*_3_ (calculated from the line intercept). (D) Secondary plot of 1/Δ_*K*s/*k*3_ *versus* 1/[I]. Δ_*K*s/*k*3_ = app *K*_s_/*k*_3 i_ – app *K*_s_/*k*_3 0_, in which app *K*_s_/*k*_3 i_ corresponds to the line in the presence of imidazole and app *K*_s_/*k*_3 0_ to the line in the absence of imidazole. (E) Secondary plot of 1/Δ_1/*k*3_ *versus* 1/[I]. Δ_1/*k*3_ = app 1/*k*_3 i_ – app 1/*k*_3 0_, in which app 1/*k*_3 i_ corresponds to the line in the presence of imidazole and app 1/*k*_3 0_ to the line in the absence of imidazole. Rates are the mean of three product determinations using the same enzyme sample. (F) Hyperbolic mixed inhibition mechanism (intersecting, hyperbolic, mixed). S, substrate NPβglc; I, inhibitor imidazole; E, enzyme Sfβgly; P, product; *K*_s_, dissociation constant of the ES complex; *K*_i_, dissociation constant of the EI complex; α*K*_i_, dissociation constant of the ESI complex; α factor represents the mutual hindering effect between S and I (α > 1); *k*_3_, rate constant of product formation complex from the ES complex; β*k*_3_, rate constant of product formation from the ESI complex, (β < 1) [1].

The mutation S247Y changed the partial competitive mechanism, originally observed for the wild‐type Sfβgly, to a linear mixed mechanism (Fig. 2). Besides that, a linear competitive mechanism was observed for the N249Q and N249A mutants (Figs 3 and 4). Finally, a partial mixed mechanism was detected for the F251A mutant (Fig. 5).

Suitable parameters for describing each imidazole inhibition mechanism were calculated based on the plots of *K*_s_/*k*_cat app_ *versus* [I] and 1/*k*_cat app_ *versus* [I] as previously established [1] (Table 1).

**Table 1:** Parameters of the imidazole inhibition mechanism for wild‐type and Sfβgly mutants. The inhibition mechanisms are depicted on Figs 2 and 5. *K*_i_ represents the dissociation constant of the Sfβgly enzyme–imidazole complex (EI). α corresponds to the mutual hindering effect involving substrate and Imidazole. α does not apply to the simple competitive mechanism. β represents the effect of imidazole on *k*_3_. β < 1 indicates that the ESI complex is less productive than the ES complex. β = 1 shows that ES and ESI are equally productive. β = 0 indicates that the ESI complex is not productive [1].

Enzyme	*K*_i_ (mm)	α	β	Mechanism
Wild‐type*	15	3	1	Partial competitive
S247Y	35	3.6	0	Linear mixed
N249A	18	–	–	Linear competitive
N249Q	39	–	–	Linear competitive
F251A	12	3	0.6	Partial mixed

*Data for the wild‐type Sfβgly are from Senger *et al*. [3].

### Mutational effects on tris inhibition

**Fig. 6:**
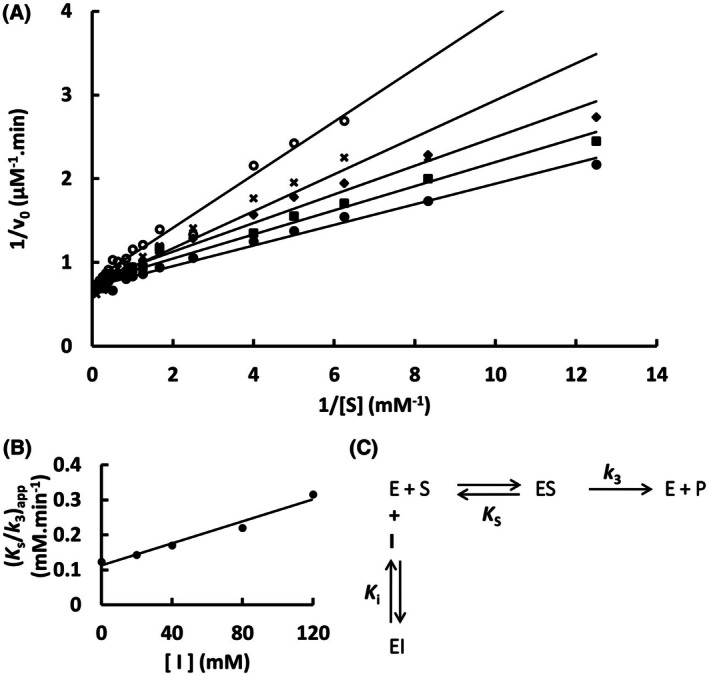
Determination of the Tris inhibition mechanism in the mutant Sfβgly S247Y. (A) Effect of different Tris concentrations (●, 0; ■, 20 mm; ◆, 40 mm; **x**, 80 mm; ○, 120 mm) on the initial rate of hydrolysis of the substrate NPβglc shown in Lineweaver–Burk plots. (B) Effect of the Tris concentration on the apparent *K*_s_/*k*_3_ (calculated from the line slope). Rates are the mean of three product determinations using the same enzyme sample. (C) Linear competitive inhibition mechanism (intersecting, linear, competitive). S, substrate NPβglc; I, inhibitor Tris; E, enzyme Sfβgly; P, product; *K*_s_, dissociation constant of the ES complex; *K*_i_, dissociation constant of the EI complex; *k*_3_, rate constant of product formation [1].

**Fig. 7:**
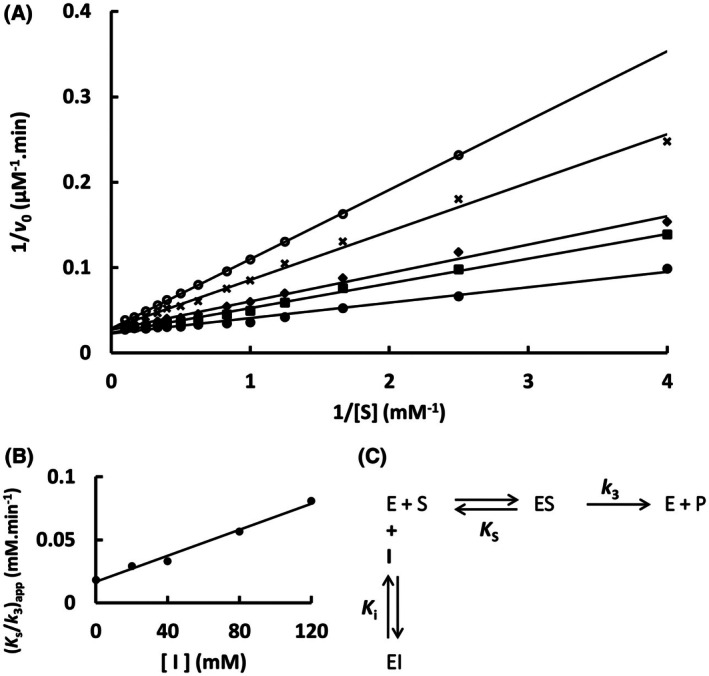
Determination of the Tris inhibition mechanism in the mutant Sfβgly N249Q. (A) Effect of different Tris concentrations (●, 0; ■, 20 mm; ◆, 40 mm; **x**, 80 mm; ○, 120 mm) on the initial rate of hydrolysis of the substrate NPβglc shown in Lineweaver–Burk plots. (B) Effect of the Tris concentration on the apparent *K*_s_/*k*_3_ (calculated from the line slope). Rates are the mean of three product determinations using the same enzyme sample. (C) Linear competitive inhibition mechanism (intersecting, linear, competitive). S, substrate NPβglc; I, inhibitor Tris; E, enzyme Sfβgly; P, product; *K*_s_, dissociation constant of the ES complex; *K*_i_, dissociation constant of the EI complex; *k*_3_, rate constant of product formation [1].

**Fig. 8:**
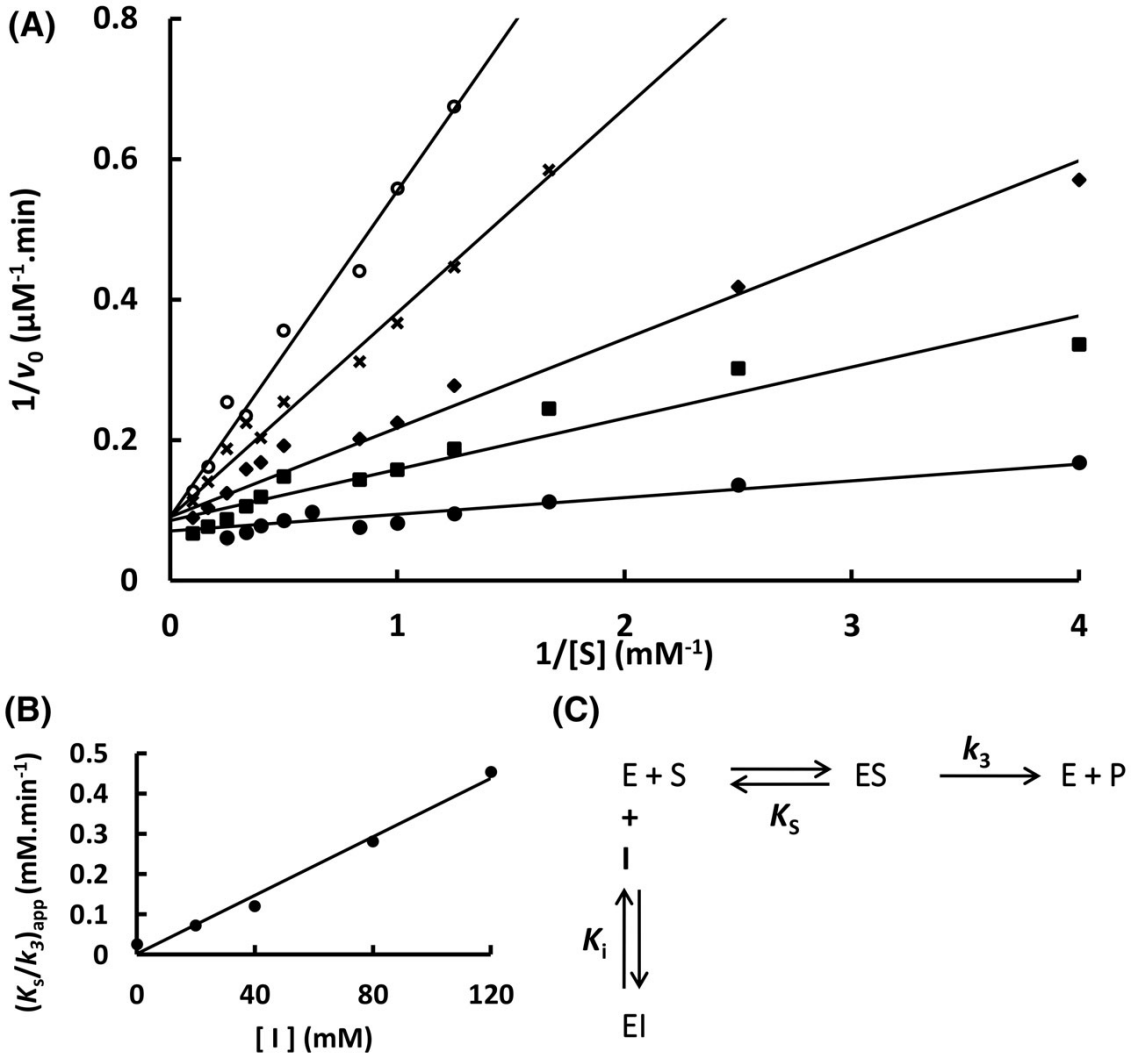
Determination of the Tris inhibition mechanism in the mutant Sfβgly N249A. (A) Effect of different Tris concentrations (●, 0; ■, 20 mm; ◆, 40 mm; **x**, 80 mm; ○, 120 mm) on the initial rate of hydrolysis of the substrate NPβglc shown in Lineweaver–Burk plots. (B) Effect of the Tris concentration on the apparent *K*_s_/*k*_3_ (calculated from the line slope). Rates are the mean of three product determinations using the same enzyme sample. (C) Linear competitive inhibition mechanism (intersecting, linear, competitive). S, substrate NPβglc; I, inhibitor Tris; E, enzyme Sfβgly; P, product; *K*_s_, dissociation constant of the ES complex; *K*_i_, dissociation constant of the EI complex; *k*_3_, rate constant of product formation [1].

The mutational effects on the Tris inhibition mechanism were less diverse. Originally, Tris was a linear mixed inhibitor of the wild‐type Sfβgly (Fig. 1). However, S247Y, N249Q and N249A mutations converted it to a linear competitive inhibitor (Figs 6, 7, 8). Thus, *K*_i_ values for Tris with these mutants were calculated based on the *K*_s_/*k*_cat app_ *versus* [I] plots [1] (Table 2).

**Table 2:** Parameters of the Tris inhibition mechanism for wild‐type and Sfβgly mutants. The inhibition mechanisms are depicted on Figs 6 and 8. *K*_i_ represents the dissociation constant of the Sfβgly enzyme‐Tris complex (EI). α corresponds to the mutual hindering effect involving substrate and Tris. α does not apply to the simple competitive mechanism. β represents the Tris effect on the *k*_cat_. β = 0 indicates that the ESI complex is not productive [1].

Enzyme	*K*_i_ (mm)	α	β	Mechanism
Wild‐type*	12	3	0	Linear mixed
S247Y	71	–	–	Linear competitive
N249A	0.6	–	–	Linear competitive
N249Q	32	–	–	Linear competitive
F251A	10	12	0	Linear mixed

*Data for the wild‐type Sfβgly are from Alvarado‐Díaz *et al*. [4].

**Fig. 9:**
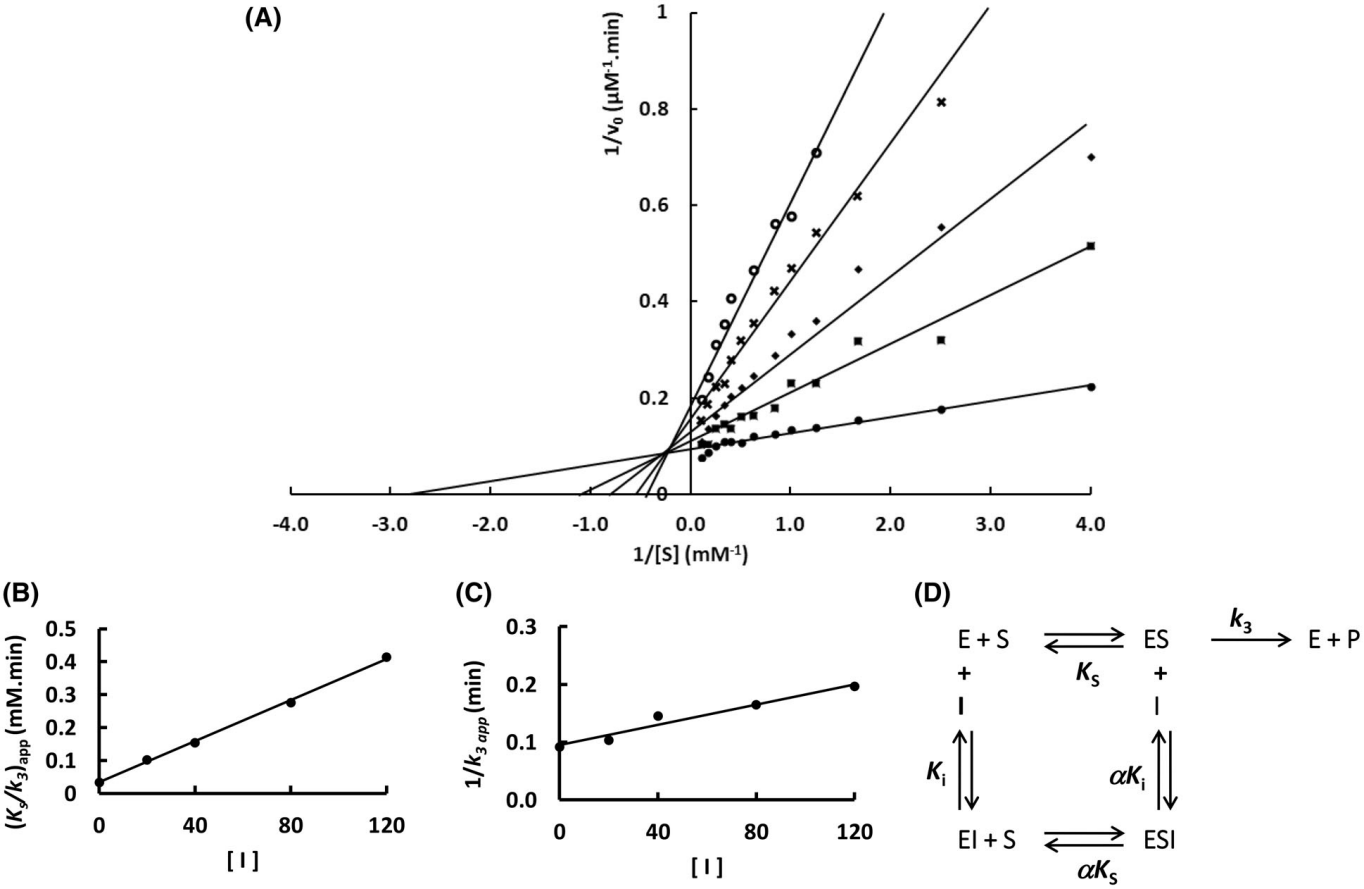
Determination of the Tris inhibition mechanism in the mutant Sfβgly F251A. (A) Effect of different Tris concentrations (●, 0; ■, 20 mm; ◆, 40 mm; **x**, 80 mm; ○, 120 mm) on the initial rate of hydrolysis of the substrate NPβglc shown in Lineweaver–Burk plots. (B) Effect of the Tris concentration on the apparent *K*_s_/*k*_3_ (calculated from the line slope). (C) Effect of the Tris concentration on the apparent 1/*k*_3_ (calculated from the line intercept). Rates are the mean of three product determinations using the same enzyme sample. (D) Linear mixed inhibition mechanism (intersecting, linear, mixed). S, substrate NPβglc; I, inhibitor Tris; E, enzyme Sfβgly; P, product; *K*_s_, dissociation constant of the ES complex; *K*_i_, dissociation constant of the EI complex; α*K*_i_, dissociation constant of the ESI complex; α, factor that represents the mutual hindering effect between S and I (α > 1); *k*_3_, rate constant of product formation [1].

Conversely, the F251A mutation had no effect on the inhibition mechanism of Tris, which remained a linear mixed type (Fig. 9). Accordingly, plots of *K*_s_/*k*_cat app_ *versus* [I] and 1/*k*_cat app_ *versus* [I] were used to determine the *K*_i_ and the α factor values for Tris and the F251A mutant [1] (Table 2).

### Mutations cause the emergence of different inhibition mechanisms

Tables 1 and 2 show a remarkable alteration of the inhibition mechanism for both imidazole and Tris because of mutations in the lateral pocket of the active site. For example, all four mutations here analyzed changed the imidazole inhibition mechanism, resulting in the emergence of three distinct mechanisms (Table 1). Besides that, three out of four mutations converted Tris into a linear competitive inhibitor of the Sfβgly (Table 2). Conversely, clear modifications in *K*_i_ are few. For example, five‐fold changes were only observed for the S247Y and N249A mutants when using Tris as an inhibitor (Table 2). Besides that, S247Y and N249Q mutations resulted in a two‐fold increase in the *K*_i_ for imidazole and Tris, respectively (Tables 1 and 2). However, given that the inhibition mechanism changed in these cases, it is not unambiguous that *K*_i_ would be describing the inhibitor interaction within the same site in the wild‐type and mutant Sfβgly.

In short, contrary to eventual expectations, the effects of the replacement in the residues that form the lateral pocket of the active site were not mainly restricted to inhibitor affinity changes. Those replacements allowed different inhibition mechanisms to emerge from those originally observed for the wild‐type Sfβgly.

This remarkable finding has to be carefully analyzed, focusing specifically on the relationship between the mechanisms observed in the wild‐type Sfβgly and those emerging in the mutants.

First, the imidazole inhibition mechanism, which is partially competitive for the wild‐type Sfβgly, changed to linear competitive in the N249A and N249Q mutants (Figs 1, 3 and 4 and Table 1). The partial competitive mechanism involves the presence of both EI and ESI complexes (Fig. 1). Hence, in the wild‐type Sfβgly, imidazole binds to a single and the same site present in both E and ES, which is compatible with the proposal that such site is the lateral pocket in the opening of the active site [8]. Moreover, imidazole binding at this site does not obstruct substrate binding in the inner region of the active site. On the other hand, the linear competitive mechanism observed for the N249A and N249Q mutants indicates the occurrence of an EI complex, in which substrate binding is impeded (Figs 3 and 4). Hence, the EI complexes present in these two mechanisms, despite being represented by the same symbol, are not physically equivalent. In the first mechanism, the imidazole interaction site within the Sfβgly is distinct from the substrate binding site, whereas, in the second mechanism, both the inhibitor and the substrate bind at the same spot. Therefore, a linear competitive inhibition mechanism is not embedded in a partial competitive one.

Next, the same comparison was drawn considering Tris. It was originally a linear mixed‐type inhibitor of the wild‐type Sfβgly (Fig. 1), which means that Tris also finds the same single binding site in E and ES, forming complexes EI and ESI. Conversely, the linear competitive inhibition observed for S247Y, N249Q and N249A mutants indicates the presence of only an EI complex, in which substrate binding is excluded (Figs 6, 7, 8 and Table 2). Hence, the EI complexes present in these two mechanisms are also not equivalent. In short, a linear competitive inhibition mechanism is not ingrained in a linear mixed one.

Therefore, considering that the emerging inhibition mechanisms mentioned above (Tables 1 and 2) were not already enclosed in those observed for the wild‐type enzyme, they could not merely result from total or partial mutational damage in the original binding site of the inhibitor. Indeed, those emerging mechanisms demand a different binding site for the inhibitor in addition to the side pocket in the active site opening. So, based on the structural and inhibition data collected with the wild‐type [7, 8, 9] and mutant Sfβgly, it is reasonable to hypothesize that more than one binding site is available to the inhibitor in the wild‐type enzyme.

### Development of a general inhibition mechanism

Hence, the simple inhibition mechanisms observed in the wild‐type and mutant Sfβgly (Fig. 1) have to be interpreted from a new perspective.

Based on this, we propose here a general inhibition mechanism featuring the same inhibitor interacting in at least two different sites.

Such a general mechanism has to adjust to the certain criteria. The different inhibitory effects, each one reflecting the inhibitor interaction in a different site, have to blend, mimicking a recognizable inhibition mechanism characterized by the inhibitor interaction within a single site. Then, these putative different inhibitory effects must merge into a single kinetic equation that describes the same behavior of the lines in the Lineweaver–Burk plot observed for single site mechanisms. Finally, such blending should be compatible with the emergence of different inhibition mechanisms. In brief, single site inhibition mechanisms have to be embedded in the multiple site mechanism.

The large number of initial possibilities to be combined in the design of such a multiple site inhibition mechanism is passable to reduction by observing the outcomes of the molecular docking of imidazole and Tris in the Sfβgly structure. Basically, imidazole and Tris are observed interacting in three different regions: the −1 subsite at the dead end of the active site, the +1 subsite closer to the active site opening and the pocket located laterally to the active site opening [8, 9] (Fig. 1). The inhibitor interactions within −1 and +1 subsites should obstruct the substrate binding, so, from that perspective, they can be seen as a single binding site. On the other hand, interactions in the lateral pocket would not interfere with the substrate binding. Therefore, the simplest mechanism combines an inhibitor interaction within the active site, which hinders the substrate binding, and an additional interaction beyond that spot with no effect on the substrate interaction.

Note that the general inhibition mechanism proposed here regards single active site enzymes presenting two binding sites for the inhibitor. So, because the substrate still has only one binding site, cooperative effects do not apply. Besides that, enzymes featuring more than two binding sites for the inhibitor are beyond the scope of this study.

**Fig. 10:**
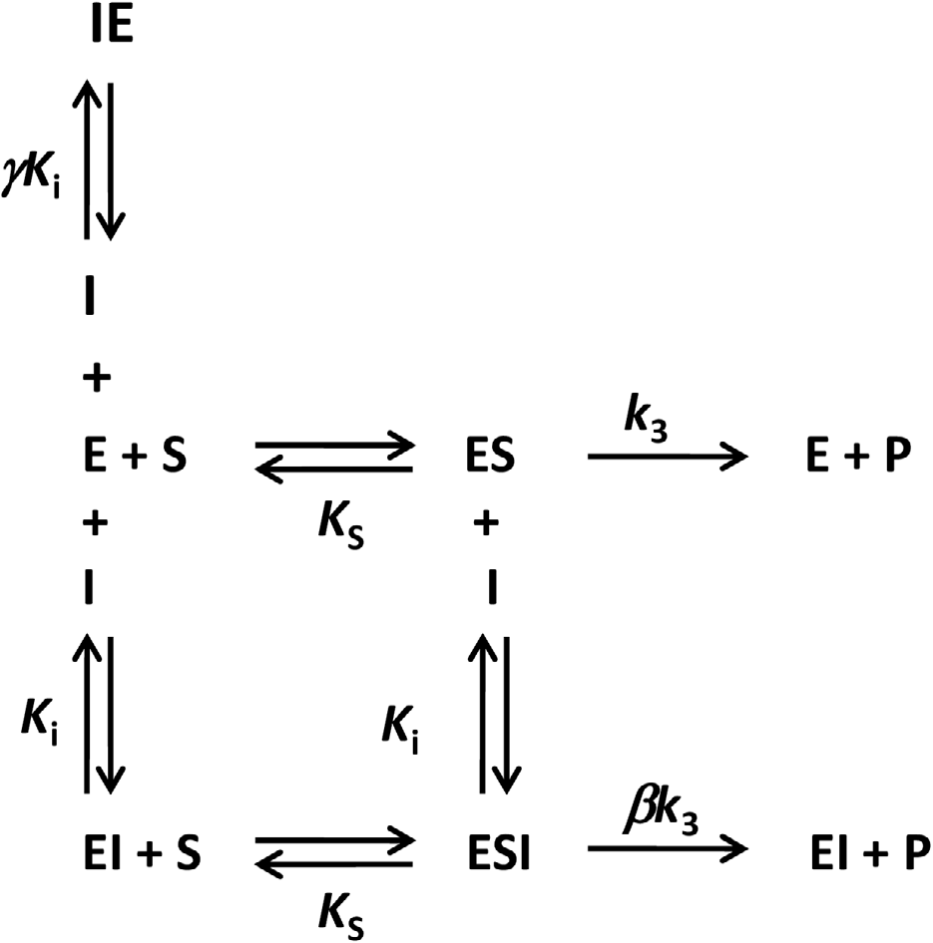
General inhibition mechanism considering two different binding sites for the inhibitor within the enzyme. One of the sites is within the active site, where the inhibitor interaction blocks the substrate binding and results in the IE complex. The second site is beyond the active site, where the inhibitor binding does not affect the substrate interaction within the active site. This binding produces the EI complex. E, free enzyme; ES, enzyme–substrate complex; IE, complex presenting the inhibitor bound within the enzyme active site; EI, complex in which the inhibitor is interacting beyond the active site; ESI, ternary complex presenting the substrate in the enzyme active site and the inhibitor bound elsewhere; γ factor represents the inhibitor relative affinity for the two binding sites available in the enzyme; β represents the inhibitor effect on the *k*_3_.

Then, in this context, Fig. 10 depicts these basic premises of the putative general inhibition mechanism. The inhibitor binding beyond the active site in the free E or ES complex produces the EI and ESI complexes; an interaction represented by the dissociation constant, *K*_i_. This inhibitor–enzyme interaction does not affect the substrate binding, so it has the same affinity for the free E and the complex EI, which is then expressed as *K*_s_. The relative reactivity of the ES and ESI complexes is expressed in the β factor. If both are equally productive, then β = 1 and *k*_3_ and β*k*_3_ are the same rate constant. Nevertheless, if ESI does not generate a product, it follows that β = 0. Finally, the range 0 < β < 1 indicates that the inhibitor hinders the product formation, so ESI is less productive than ES (β*k*_3_ < *k*_3_). In addition to that, inhibitor binding within the active site *per se* forms the IE complex, an equilibrium mediated by the γ*K*_i_ dissociation constant. The factor γ multiplying *K*_i_ was introduced to express the relative affinity of the same inhibitor for these two different sites, i.e., the region beyond the active site and that within the active site. Thus, if γ = 1, the inhibitor presents the same affinity for both sites. Conversely, γ < 1 indicates that the inhibitor binds preferentially in the active site. An extreme value, γ << 1, points to an exclusive interaction within the active site. The opposite is true for γ factor higher than 1.

Based on these premises, a general kinetic equation expressing the effect of the [S] on the initial reaction rate (*v*_0_) in the presence of a [I] was deduced (Eqn 1) (Fig. S3):

(1) $$\frac{1}{v_{0}}=\frac{K_{s}}{V_{\max}}\frac{\left(1+\frac{\left[I\right]}{{\mathrm{\gamma K}}_{i}}+\frac{\left[I\right]}{K_{i}}\right)}{\left(1+\frac{\beta \left[I\right]}{K_{i}}\right)}\;\frac{1}{\left[S\right]}+\frac{1}{V_{\max}}\frac{\left(1+\frac{\left[I\right]}{K_{i}}\right)}{\left(1+\frac{\beta \left[I\right]}{K_{i}}\right)}$$

### The general model explains the imidazole and tris inhibition of the wild‐type sfβgly

As mentioned above, if the general inhibition model is correct, even presenting two binding sites for the same I, it should result in a kinetic equation that describes the lines in the Lineweaver–Burk plot that have the same behavior as those observed for single site mechanisms. Hence, taking into account our working model here, the general inhibition model [Fig. 10 and Eqn (1)] has to produce data that could be interpreted as partial competitive and linear mixed mechanisms, which were observed when imidazole and Tris inhibited the wild‐type Sfβgly, respectively (Tables 1 and 2) [8, 9].

Hence assuming a particular situation in which the inhibitor is totally innocuous regarding the product formation, β = 1, Eqn (1) simplifies to Eqn (2) (Fig. S3):

(2) $$\frac{1}{v_{0}}=\frac{K_{s}}{V_{\max}}\frac{\left(1+\frac{\left[I\right]}{{\mathrm{\gamma K}}_{i}}+\frac{\left[I\right]}{K_{i}}\right)}{\left(1+\frac{\left[I\right]}{K_{i}}\right)}\;\frac{1}{\left[S\right]}+\frac{1}{V_{\max}}$$

Equation (2) shows an intercept $\left(\frac{1}{V_{\max}}\right)$ that does not depend on [I]. So, by assuming equally productive ES and ESI (β = 1), the general inhibition mechanism (Fig. 10) generates a *v*_0_ dependence on [S] and [I] that in the 1/*v*_0_ *versus* 1/[S] plot results in lines that converge to the same point on the *y*‐axis regardless of [I]. Besides that, the slope of those lines (*K*_s_/*V*_max app_) increases up to a limiting value, $\left(1+\frac{1}{\gamma }\right)$, at infinite [I], producing a hyperbolic curve in the *K*_s_/*V*_max app_ *versus* [I] plot (Fig. S3). These are the same features of a partial competitive inhibitor, as observed for the inhibitory effect of imidazole on the wild‐type Sfβgly (Fig. 1) [8].

Furthermore, when the general model (Fig. 10) is interpreted by assuming β = 0, which indicates an ESI complex totally unproductive, the general Eqn (1) is reduced to the form presented in Eqn (3) (Fig. S3):

(3) $$\frac{1}{v_{0}}=\frac{K_{s}}{V_{\max}}\left(1+\frac{\left[I\right]}{{\mathrm{\gamma K}}_{i}}+\frac{\left[I\right]}{K_{i}}\right)\;\frac{1}{\left[S\right]}+\frac{1}{V_{\max}}\left(1+\frac{\left[I\right]}{K_{i}}\right)$$

Thus, Eqn (3) shows that, if the ESI complex does not form product (β = 0), the general inhibition mechanism generates a *v*_0_ dependence on [S] and [I] that, in the Lineweaver–Burk plot produces lines that crosses at the second quadrant, whereas their slopes (*K*_s_/*V*_max app_) and intercepts (1/*V*_max app_) show a linear growth in the presence of increasing [I] (Fig. S3). These are the properties of a linear mixed inhibitor, as Tris is to the wild‐type Sfβgly (Fig. 1) [1].

Therefore, the previously detected inhibition mechanisms for imidazole and Tris, partial competitive and linear mixed, respectively, which were traditionally understood as a single binding site mechanism, can indeed arise from the same inhibitor interaction within two different sites following the premises expressed in the general model here presented (Fig. 10).

### The general model explains the imidazole and Tris inhibition of the mutant sfβgly

As pointed out above, the general inhibition model, even presenting two binding sites for the same I, should be compatible with the emergence of different simple inhibition mechanisms.

In this context, recalling that the scheme presented in Fig. 10 was designed as a general model, one could then assume a condition not discussed yet (i.e. 0 < β < 1). In this situation, in which the ESI complex is less productive than the ES, the general Eqn (1) is applied without modifications. In that format, Eqn (1) indicates that lines in the Lineweaver–Burk plot cross in the second quadrant, whereas their slopes (*K*_s_/*V*_max app_) and intercepts (1/*V*_max app_) show a hyperbolic growth in the presence of increasing [I], reaching plateaus at infinite [I], $\left(1+\frac{1}{\gamma }\right)$ and $\left(\frac{1\;}{\beta }\right)$, respectively (Fig. S3). Those are the features of a partial mixed inhibitor, as observed when using imidazole as an inhibitor of the F251A mutant (Fig. 5 and Table 1). Hence, the effect of the F251A replacement seems to be a modification of the β factor from 1 to 0.6 (Table 1), which in the context of the general Eqn (1) is enough to generate a partial mixed mechanism from a partial competitive one, previously seen for the wild‐type (Figs 1 and 5 and Table 1).

A similar discussion can be elaborated regarding the inhibition of the S247Y mutant by Imidazole (Fig. 2 and Table 1). As mentioned above, when the β factor drops to 0, this modification converts the general Eqn (1) from a partial competitive (β = 1) to linear mixed (β = 0) (Eqn 3). So, the effect of the mutation S247Y seems to be the inactivation of the ESI complex involving Imidazole, which produced a linear mixed mechanism from a partial competitive one.

Thus, the general model (Fig. 10) (Eqn 1) also applies to understand the mutational effects on the emergence of different inhibition mechanisms from those observed in the wild‐type Sfβgly. Particularly as presented above, different mechanisms, partial competitive, partial mixed and linear mixed, interconvert simply by modifications in the reactivity of the ESI complex, expressed in the β factor that multiplies *k*_3_. So, we hypothesize that mutations F251A and S247Y may have changed the imidazole interactions and positioning in the lateral pocket of the Sfβgly active site, which triggered an alteration in the nearby interactions of the substrate transition state within the active site, consequently decreasing the stability of the ESI complex, which was manifested in *k*_3_ variations expressed by the β factor. That agrees with the understanding that enzymes preferentially bind the transition state of the substrate [19].

Now, to complete the analysis of how the general inhibition model explains the emergence of the different inhibition mechanisms upon mutation of the Sfβgly, it remains to approach the N249A and N249Q mutants in the presence of imidazole and S247Y, N249A and N249Q mutants in the presence of Tris (Tables 1 and 2). In these cases, a linear competitive mechanism was systematically detected.

A qualitative inspection of the general model (Fig. 10) suggests that, if an inhibitor binds more strongly within the active site, forming the IE complex, and the inhibitor concentration is lower than the dissociation constant of the second binding site (outside the active site), the relative concentration of the complexes EI and ESI will be insignificant. This situation can be quantitatively approached by setting γ << 1 and β = 1 when [I] < *K*_i_ in the general Eqn (1), which is simplified to Eqn (4) (Fig. S3):

(4) $$\frac{1}{v_{0}}=\frac{K_{s}}{V_{\max}}\left(\frac{1}{{\mathrm{\gamma K}}_{i}}\left[I\right]\right)\frac{1}{\left[S\right]}+\frac{1}{V_{\max}}$$

Such a combination, γ << 1 and β = 1, suggests that the mutations N249A and N249Q (Table 1) and also S247Y, N249A and N249Q (Table 2) basically disrupted imidazole and Tris interactions around the Sfβgly active site opening, making the binding in the interior of the active site relatively much more favorable.

Finally, recalling again that the scheme presented in Fig. 10 was designed as a general model, one could then analyze combinations of β and γ factors not discussed yet. For example, assuming β = 0 and γ >> 1, which means an unproductive ESI complex and higher inhibitor affinity for the binding site around the active site opening, the Eqn (3) above simplifies to the Eqn (5) (Fig. S3):

(5) $$\frac{1}{v_{0}}=\frac{K_{s}}{V_{\max}}\left(1+\frac{\left[I\right]}{K_{i}}\right)\;\frac{1}{\left[S\right]}+\frac{1}{V_{\max}}\left(1+\frac{\left[I\right]}{K_{i}}\right)$$

As seen, Eqn (5) describes a linear non‐competitive inhibition mechanism (intersecting, linear, non‐competitive) [1], characterized by lines in the Lineweaver–Burk plot with (*K*_s_/*V*_max app_) and (1/*V*_max app_) showing a linear growth in the presence of increasing [I] (Fig. S3). Besides that, lines observed at different [I] crosses at the same point in the 1/[S] axis, as shown by the same term multiplying both the slope and intercept in Eqn (5).

A final combination is 0 < β < 1 and γ >> 1, which means an ESI less productive than the ES complex combined to a higher inhibitor affinity for the binding site beyond the active site. In that condition, Eqn (1) transforms into Eqn (6) (Fig. S3):

(6) $$\frac{1}{v_{0}}=\frac{K_{s}}{V_{\max}}\frac{\left(1+\frac{\left[I\right]}{K_{i}}\right)}{\left(1+\frac{\beta \left[I\right]}{K_{i}}\right)}\;\frac{1}{\left[S\right]}+\frac{1}{V_{\max}}\frac{\left(1+\frac{\left[I\right]}{K_{i}}\right)}{\left(1+\frac{\beta \left[I\right]}{K_{i}}\right)}$$

Equation (6) describes lines in a Lineweaver–Burk plot that present increasing slopes and intercepts as the [I] rises. In addition, such increment is hyperbolic and at infinite [I] both parameters reach a limiting plateau equal to $\left(\frac{1\;}{\beta }\right)$ (Fig. S3). Those are features of partial non‐competitive inhibitors (intersecting, hyperbolic, non‐competitive; [1]).

Then, considering these two last theoretical situations and those discussed above regarding the mutant and wild‐type Sfβgly, the foundations of the general inhibition model (Fig. 10) engendered six different simple inhibition mechanisms (three linear and three hyperbolic or partial). In conclusion, the inhibition model proposed here met the criteria for a general mechanism that is based on a double binding site mimicking simple inhibition mechanisms.

### The γ factor explains the apparent hindrances between I and S in the formation of the ESI complex

Notably, the presence of the γ factor confers interesting features to the general inhibition model (Fig. 10). For example, considering the comparison of two situations, γ < 1 and γ = 1, in the former, a larger part of the enzyme population is diverted to the IE complex, and lower concentrations of the ESI and ES complexes are observed in the same [S] and [I]. Consequently, the presence of the IE complex introduces an effect of apparent *K*_i_ and *K*_s_ increments because higher [I] and [S] will be needed to reach the same levels observed for the situation in which γ = 1. Traditionally, such apparent *K*_i_ and *K*_s_ increments observed in inhibition mechanisms that involve an ESI complex are interpreted as a mutual hindrance between S and I in the formation of the ESI complex. To account for that, an α factor (α > 1) is usually introduced multiplying the *K*_i_ and *K*_s_ in those kinetic models and equations [1].

Nevertheless, here, we propose that the mutual hindrance between S and I is just an apparent effect because of the carriage of part of the enzyme population to the IE complex, which goes back to a clear affinity difference between the two binding sites of the inhibitor. Consequently, the traditional α factor can be expressed in terms of the γ factor (Eqn 5) (Fig. S3).

(7) $$\alpha =\frac{1}{\gamma }+1$$

This inverse relation indicates that as the inhibitor affinity for the active site becomes dominant, which is represented by the γ factor dropping below 1, the α factor becomes higher than 1. Hence, an apparent mutual hindrance between S and I is observed (Fig. S3). Oppositely, as the inhibitor preferentially binds elsewhere beyond the active site, the γ factor increases, getting higher than 1, and, as a consequence, α tends to 1. So, the apparent hindrance disappears.

In brief, no physical obstruction between S and I occurs in the formation of ESI complexes. Indeed, the apparent hindrance between S and I emerges from the inhibitor relative affinity for two independent binding sites.

Based on that, Tables 1 and 2 should be reinterpreted by converting the α in the γ factor, using Eqn (5) (Table 3). In brief, γ factors indicate that both inhibitors have a two‐fold higher affinity for the active site of the wild‐type Sfβgly than for the binding in the side pocket of the active site opening. In addition to that, based on Eqns (1) and (2) (above), the dissociation constants of the inhibitor in the active site (γ*K*_i_) and in the pocket around its opening (*K*_i_) were calculated based on the terms multiplying the *K*_s_/*V*_max_ as described in the Fig. S4. Thus, the γ*K*_i_ and *K*_i_ were respectively estimated as 22.5 and 45 mm for imidazole and 18 and 36 mm for Tris, confirming that both inhibitors have higher affinity for the active site.

**Table 3:** Relative binding affinity of inhibitors within and outside the active site of Sfβgly. The γ factor < 1 indicates that the inhibitor has a higher binding affinity in the active site than around the active site opening. The opposite holds for γ factor > 1.

Inhibitor	Enzyme	γ factor
Imidazole	Wild‐type*	0.5
	S247Y	0.4
	F251A	0.5
Tris	Wild‐type*	0.5
	F251A	0.09

*Calculated based on the references [8] and [9], respectively.

Finally, this is an interesting contribution of the application of the general inhibition model to interpret enzyme inhibition data because it revealed that inhibitors that were assumed to interact only outside the active site actually have higher affinity for the active site. Such information may be valuable when screening libraries of potential inhibitors.

## Conclusions

The general inhibition model depicted in Fig. 10 and Eqn (1) is robust enough to be the foundation for the emergence of the complete set of inhibition mechanisms shown in Fig. S1 and Tables 1 and 2. The two interaction sites for the inhibitor featured in the model agree with the structural data (crystallographic and computational) showing Tris and imidazole binding in two different regions: within the active site and around the active site opening [8, 9]. Moreover, the relative affinity of Tris for these two sites, calculated using the general equation, shows a preferential binding in the active site, which agrees with the crystallographic data [7]. Modifications of the β factor generate partial competitive, partial mixed and linear mixed inhibition mechanisms. The β factor changes merely reflect differences in the ESI reactivity, which is in line with the perspective that enzymes preferentially bind the substrate transition state [19]. Besides that, combined modifications of the γ and β factors generate linear competitive, linear non‐competitive and partial non‐competitive inhibition mechanisms.

In brief, the general model not only explains the inhibition data presented here for the wild‐type and mutant Sfβgly, but also brings together, on top of the same theoretical foundation, six fundamental inhibition mechanisms previously described in the literature.

The statement that small molecule inhibitors may find more than one binding site in an enzyme structure is realistic. A range of relative affinities for those sites, represented in the γ factor, is a natural consequence. The assumption that inhibitor and enzyme interactions may be manifested in a range of effects on the enzyme interactions with the substrate transition state, which is encapsulated in the β factor, is also reasonable. Therefore, the general inhibition model (Fig. 10) (Eqn 1) is a realistic perspective of the interaction of small molecule inhibitors with enzymes, explaining a broad range of phenomena previously attributed to diverse mechanisms.

In short, we conclude that six fundamental inhibition mechanisms, linear and hyperbolic, are facets of the same general inhibition model presented here.

## Conflicts of interest

The authors declare that they have no conflicts of interest.

## Author contributions

RSC and SRM were both responsible for the conceptualization, formal analysis, investigations, writing the original draft and reviewing the final version of the manuscript. In addition, SRM was also responsible for funding acquisition and resources.

## Supporting information


**Fig. S1.** Simple and intercepting inhibition mechanisms. Simple mechanisms are those in which only one inhibitor molecule binds in a single site in the enzyme. Intercepting mechanisms are those in which lines are observed in the plots of *K*_s_/*k*_cat app_ *versus* [I] and 1/*k*_cat app_ *versus* [I].

**Fig. S2.** SDS/PAGE of the purified recombinant Sfβgly mutants.

**Fig. S3.** General mechanism of enzyme inhibition: equations and deductions.

**Fig. S4.** Determination of *K*_i_ using the general inhibition model.

**Data S1.** Data used in the determination of the imidazole and Tris inhibition mechanisms of the mutant Sfβgly.

## Data Availability

The data that support the findings of this study are available in the Supporting information.
