# Supplementary figures and images for: A general model for analysis of linear and hyperbolic enzyme inhibition mechanisms
Source: FEBS Open Bio. 2025 Sep 24;16(2):365–81. doi: 10.1002/2211-5463.70128 (PMC12871559; doi:10.1002/2211-5463.70128)

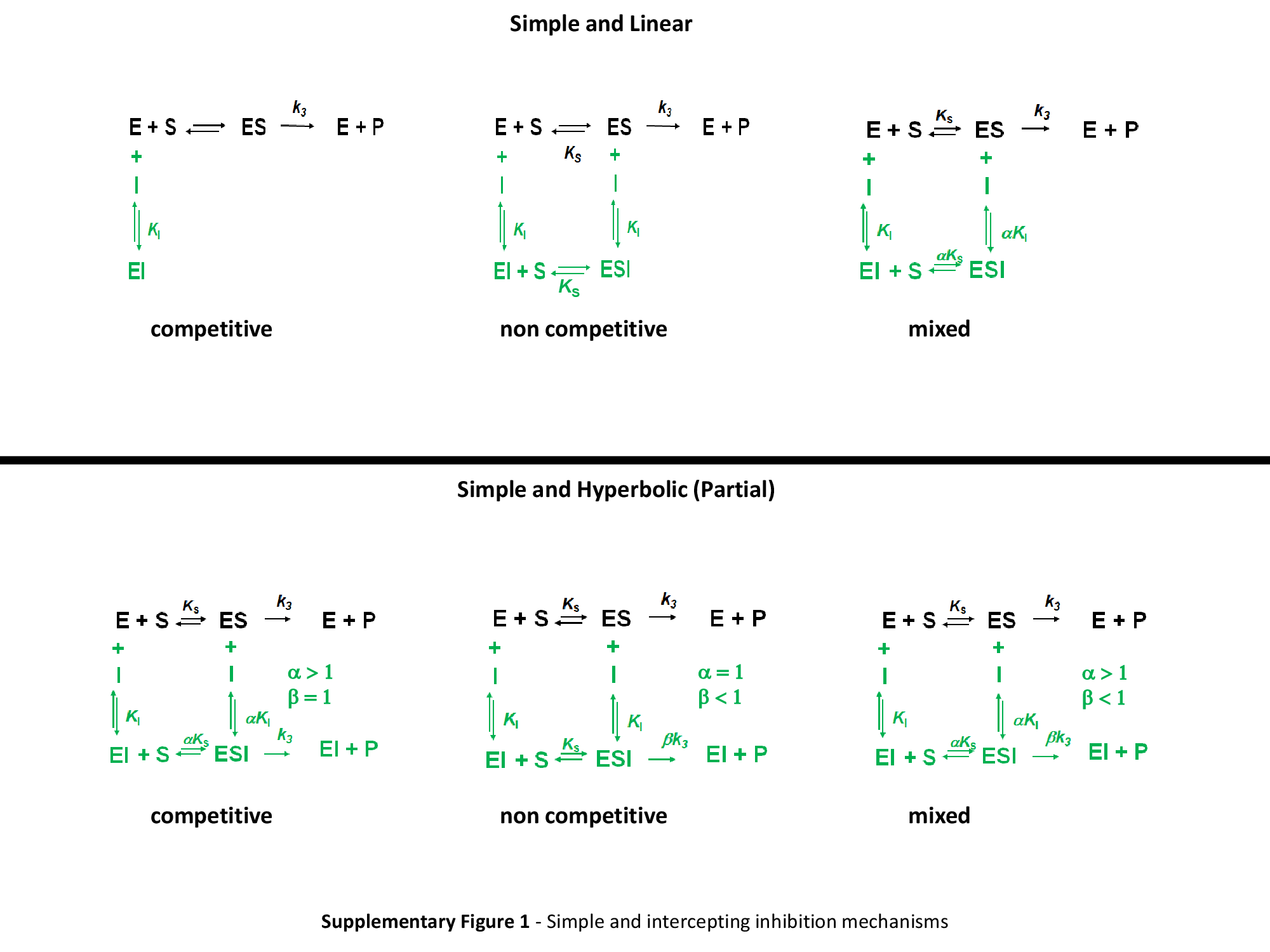

Supplement: Supplementary file 1 — Fig. S1. Simple and intercepting inhibition mechanisms. Simple mechanisms are those in which only one inhibitor molecule binds in a single site in the enzyme. Intercepting mechanisms are those in which lines are observed in the plots of K s/k cat app versus [I] and 1/k cat app versus [I]. [file FEB4-16-365-s001.tiff]
