# Supplementary material for: A general model for analysis of linear and hyperbolic enzyme inhibition mechanisms
Source: FEBS Open Bio. 2025 Sep 24;16(2):365–81. doi: 10.1002/2211-5463.70128 (PMC12871559; doi:10.1002/2211-5463.70128)
Supplement: Supplementary file 3 — Fig. S3. General mechanism of enzyme inhibition: equations and deductions. [file FEB4-16-365-s004.pdf]

Supplementary Figure 3 – General Mechanism of Enzyme Inhibition: Equations and Deductions

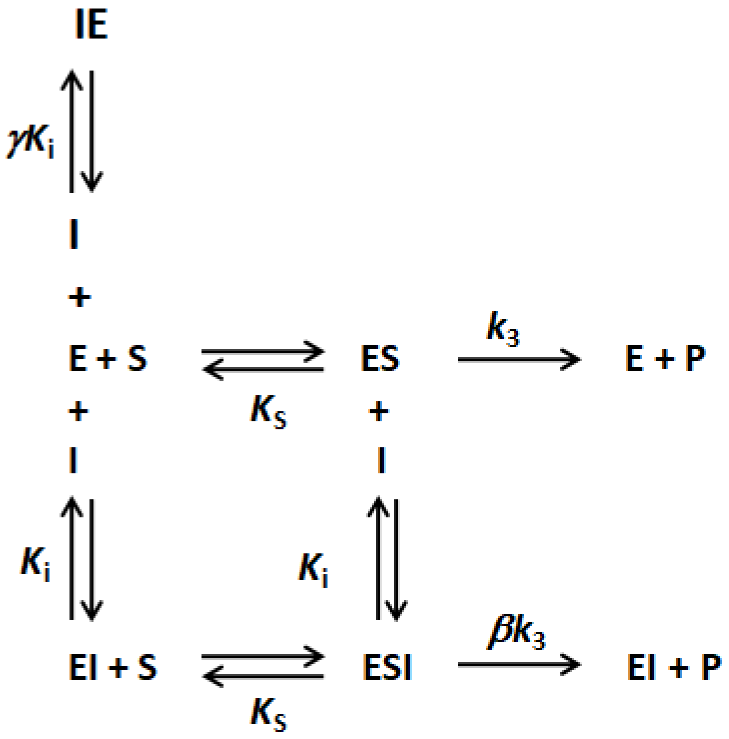

Inhibition mechanism based on two different binding sites of the inhibitor in the enzyme. One of the sites is within the active site, where the inhibitor interaction blocks the substrate binding and results in the IE complex. The second site is beyond the active site, where the inhibitor binding does not affect the substrate interaction within the active site. Inhibitor binding in this second site produces the EI complex. E, free enzyme; ES, enzyme-substrate complex; IE, complex presenting the inhibitor bound within the enzyme active site; EI, complex in which the inhibitor is interacting beyond the active site; ESI, ternary complex presenting the substrate in the enzyme active site and the inhibitor bound elsewhere; γ factor represents the inhibitor relative affinity for the two binding sites available in the enzyme; β represents the inhibitor effect on the k_3.

Based on this scheme, the dissociation constants and the respective complexes are defined as seem below.

$$K_S = \frac{[E][S]}{[ES]} \qquad [ES] = \frac{[E][S]}{K_S}$$

$$K_S = \frac{[EI][S]}{[ESI]} \qquad [ESI] = \frac{[EI][S]}{K_S} = \frac{[E][I][S]}{K_S K_i}$$

$$K_i = \frac{[E][I]}{[EI]} \qquad [EI] = \frac{[E][I]}{K_i}$$

$$\gamma K_i = \frac{[E][I]}{[IE]} \qquad [IE] = \frac{[E][I]}{\gamma K_i}$$

The initial (v_0) and maximum rate (V_{max}) are expressed as:

$$v_0 \; = \; k_3[ES] + \beta k_3[ESI]$$

$$V_{max} \; = \; k_3[E]_{total}$$

The total enzyme population ($[E]_{total}$) is distributed among the species presented in the scheme above.

$$[E]_{total} = [IE] + \; [E] \; + [EI] + \; [ES] + [ESI]$$

Based on these definitions the relative rate v_0/V_{max} is expressed as:

$$\frac{v_0}{V_{max}} = \frac{k_3[ES] + \beta k_3[ESI]}{k_3([IE] + \; [E] \; + [EI] + \; [ES] + [ESI])}$$

Definitions of the complexes ES, ESI, EI, IE e ESI are inserted into the v_0/V_{max} equation.

$$\frac{v_0}{V_{max}} = \frac{k_3 \frac{[E][S]}{K_S} + \beta k_3 \frac{[E][I][S]}{K_S K_i}}{k_3 \left(\frac{[E][I]}{\gamma K_i} + [E] \; + \frac{[E][I]}{K_i} + \; \frac{[E][S]}{K_S} + \frac{[E][I][S]}{K_S K_i} \right)}$$

The equation above is simplified by eliminating k_3 and [E]

$$\frac{v_0}{V_{max}} = \frac{\frac{[S]}{K_S} + \frac{\beta[I][S]}{K_S K_i}}{\left(\frac{[I]}{\gamma K_i} + 1 + \frac{[I]}{K_i} + \frac{[S]}{K_S} + \frac{[I][S]}{K_S K_i}\right)}$$

$$\frac{v_0}{V_{max}} = \frac{\frac{[S]}{K_S}\left(1 + \frac{\beta[I]}{K_i}\right)}{\left(\frac{[I]}{\gamma K_i} + 1 + \frac{[I]}{K_i} + \frac{[S]}{K_S} + \frac{[I][S]}{K_S K_i}\right)}$$

Numerator and denominator are multiplied by K_s.

$$\frac{v_0}{V_{max}} = \frac{[S]\left(1 + \frac{\beta[I]}{K_i}\right)}{\left(\frac{K_S[I]}{\gamma K_i} + K_S + \frac{K_S[I]}{K_i} + [S] + \frac{[I][S]}{K_i}\right)}$$

In the denominator, find a common factor in the terms containing K_s and [S].

$$\frac{v_0}{V_{max}} = \frac{[S]\left(1 + \frac{\beta[I]}{K_i}\right)}{K_S\left(1 + \frac{[I]}{\gamma K_i} + \frac{[I]}{K_i}\right) + [S]\left(1 + \frac{[I]}{K_i}\right)}$$

Numerator and denominator are divided by $\left(1 + \frac{\beta[I]}{K_i}\right)$.

$$\frac{v_0}{V_{max}} = \frac{[S]}{K_S\left(\frac{1 + \frac{[I]}{\gamma K_i} + \frac{[I]}{K_i}}{1 + \frac{\beta[I]}{K_i}}\right) + [S]\left(\frac{1 + \frac{[I]}{K_i}}{1 + \frac{\beta[I]}{K_i}}\right)}$$

$$v_0 = \frac{V_{max}[S]}{K_S\left(\frac{1 + \frac{[I]}{\gamma K_i} + \frac{[I]}{K_i}}{1 + \frac{\beta[I]}{K_i}}\right) + [S]\left(\frac{1 + \frac{[I]}{K_i}}{1 + \frac{\beta[I]}{K_i}}\right)}$$

Equation 1

The equation 1 is similar to the Michaelis-Menten equation. Hence, it is linearized using the Lineweaver-Burk inversion.

$$\frac{1}{v_0} = \frac{K_S}{V_{max}}\left(\frac{1 + \frac{[I]}{\gamma K_i} + \frac{[I]}{K_i}}{1 + \frac{\beta[I]}{K_i}}\right)\frac{1}{[S]} + \frac{1}{V_{max}}\left(\frac{1 + \frac{[I]}{K_i}}{1 + \frac{\beta[I]}{K_i}}\right)$$

Equation 2

Case 1 – General Inhibition Model in β = 1

Taking the general inhibition model above and assuming β = 1, the dissociation constants and the respective complexes are defined as:

$$K_S = \frac{[E][S]}{[ES]} \qquad [ES] = \frac{[E][S]}{K_S}$$

$$K_S = \frac{[EI][S]}{[ESI]} \qquad [ESI] = \frac{[EI][S]}{K_S} = \frac{[E][I][S]}{K_S K_i}$$

$$K_i = \frac{[E][I]}{[EI]} \qquad [EI] = \frac{[E][I]}{K_i}$$

$$\gamma K_i = \frac{[E][I]}{[IE]} \qquad [IE] = \frac{[E][I]}{\gamma K_i}$$

The initial (v_0) and maximum rate (V_{max}) are expressed as:

$$v_0 \; = \; k_3[ES] + k_3[ESI]$$

$$V_{max} \; = \; k_3[E]_{total}$$

The total enzyme population ($[E]_{total}$) is distributed among the species presented in the scheme above.

$$[E]_{total} = [IE] + \; [E] \; + [EI] + \; [ES] + [ESI]$$

Based on these definitions the relative rate v_0/V_{max} is expressed as:

$$\frac{v_0}{V_{max}} = \frac{k_3[ES] + k_3[ESI]}{k_3([IE] + \; [E] \; + [EI] + \; [ES] + [ESI])}$$

Definitions of the complexes ES, ESI, EI, IE e ESI are inserted into the v_0/V_{max} equation.

$$\frac{v_0}{V_{max}} = \frac{k_3\left(\frac{[E][S]}{K_S} + \frac{[E][I][S]}{K_S K_i}\right)}{k_3\left(\frac{[E][I]}{\gamma K_i} + [E] \; + \frac{[E][I]}{K_i} + \; \frac{[E][S]}{K_S} + \frac{[E][I][S]}{K_S K_i}\right)}$$

The equation above is simplified by eliminating k_3 and [E]

$$\frac{v_0}{V_{max}} = \frac{\frac{[S]}{K_S} + \frac{[I][S]}{K_S K_i}}{\left(\frac{[I]}{\gamma K_i} + 1 + \frac{[I]}{K_i} + \frac{[S]}{K_S} + \frac{[I][S]}{K_S K_i}\right)}$$

$$\frac{v_0}{V_{max}} = \frac{\frac{[S]}{K_S}\left(1 + \frac{[I]}{K_i}\right)}{\left(\frac{[I]}{\gamma K_i} + 1 + \frac{[I]}{K_i} + \frac{[S]}{K_S} + \frac{[I][S]}{K_S K_i}\right)}$$

Numerator and denominator are multiplied by K_s.

$$\frac{v_0}{V_{max}} = \frac{[S]\left(1 + \frac{[I]}{K_i}\right)}{\left(\frac{K_S[I]}{\gamma K_i} + K_S + \frac{K_S[I]}{K_i} + [S] + \frac{[I][S]}{K_i}\right)}$$

In the denominator, find a common factor in the terms containing K_s and [S].

$$\frac{v_0}{V_{max}} = \frac{[S]\left(1 + \frac{[I]}{K_i}\right)}{K_S\left(1 + \frac{[I]}{\gamma K_i} + \frac{[I]}{K_i}\right) + [S]\left(1 + \frac{[I]}{K_i}\right)}$$

Numerator and denominator are divided by $\left(1 + \frac{[I]}{K_i}\right)$.

$$\frac{v_0}{V_{max}} = \frac{[S]}{K_S\left(\frac{1 + \frac{[I]}{\gamma K_i} + \frac{[I]}{K_i}}{1 + \frac{[I]}{K_i}}\right) + [S]\left(\frac{1 + \frac{[I]}{K_i}}{1 + \frac{[I]}{K_i}}\right)}$$

$$v_0 = \frac{V_{max}[S]}{K_S\left(\frac{1 + \frac{[I]}{\gamma K_i} + \frac{[I]}{K_i}}{1 + \frac{[I]}{K_i}}\right) + [S]}$$

Equation 3

The equation 3 is similar to the Michaelis-Menten equation. Hence, it is linearized using the Lineweaver-Burk inversion.

$$\frac{1}{v_0} = \frac{K_S}{V_{max}}\left(\frac{1 + \frac{[I]}{\gamma K_i} + \frac{[I]}{K_i}}{1 + \frac{[I]}{K_i}}\right)\frac{1}{[S]} + \frac{1}{V_{max}}$$

Equation 4

Based on the equation 4, by isolating the term that multiplies the slope (K_s/V_{max}), we can analyze the behavior of this term as a function of [I].

$$multiplicative\ term = \frac{\left(1 + \frac{[I]}{\gamma K_i} + \frac{[I]}{K_i}\right)}{\left(1 + \frac{[I]}{K_i}\right)}$$

$$if\ [I] \rightarrow \infty\ \therefore\ \frac{[I]}{\gamma K_i} + \frac{[I]}{K_i} \gg 1\ and\ \frac{[I]}{K_i} \gg 1$$

$$\frac{\left(1 + \frac{[I]}{\gamma K_i} + \frac{[I]}{K_i}\right)}{\left(1 + \frac{[I]}{K_i}\right)} = \frac{\left(\frac{[I]}{\gamma K_i} + \frac{[I]}{K_i}\right)}{\left(\frac{[I]}{K_i}\right)} = \frac{\frac{[I]}{K_i}\left(1 + \frac{1}{\gamma}\right)}{\left(\frac{[I]}{K_i}\right)} = 1 + \frac{1}{\gamma}$$

Thus, the multiplicative term has a finite and constant value at infinite [I].
In addition to that, we can simulate the behavior of this term as a function of [I].

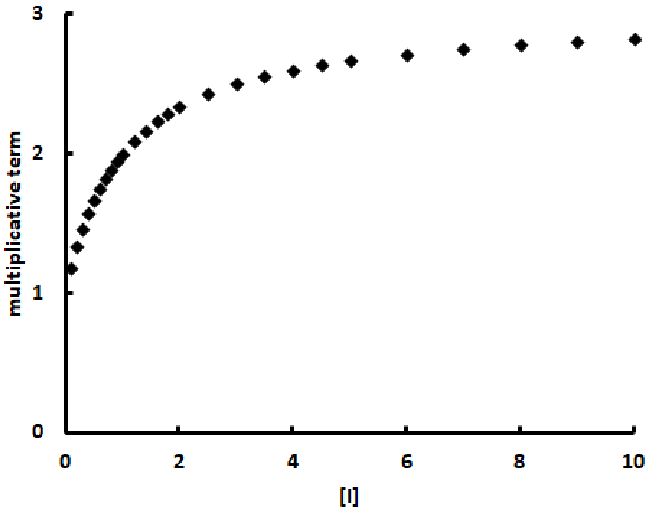

Simulation of the multiplicative term magnitude as a function of [I]. K_i = 1 and γ = 0.5

Case 2 – General Inhibition Model in β = 0

Taking the general inhibition model above and assuming β = 0, the dissociation constants and the respective complexes are defined as:

$$K_S = \frac{[E][S]}{[ES]} \qquad [ES] = \frac{[E][S]}{K_S}$$

$$K_S = \frac{[EI][S]}{[ESI]} \qquad [ESI] = \frac{[EI][S]}{K_S} = \frac{[E][I][S]}{K_S K_i}$$

$$K_i = \frac{[E][I]}{[EI]} \qquad [EI] = \frac{[E][I]}{K_i}$$

$$\gamma K_i = \frac{[E][I]}{[IE]} \qquad [IE] = \frac{[E][I]}{\gamma K_i}$$

The initial (v_0) and maximum rate (V_{max}) are expressed as:

$$v_0 = k_3[ES]$$

$$V_{max} = k_3[E]_{total}$$

The total enzyme population ($[E]_{total}$) is distributed among the species presented in the scheme above.

$$[E]_{total} = [IE] + [E] + [EI] + [ES] + [ESI]$$

Based on these definitions the relative rate v_0/V_{max} is expressed as:

$$\frac{v_0}{V_{max}} = \frac{k_3[ES]}{k_3([IE] + [E] + [EI] + [ES] + [ESI])}$$

Definitions of the complexes ES, ESI, EI, IE e ESI are inserted into the v_0/V_{max} equation.

$$\frac{v_0}{V_{max}} = \frac{k_3\left(\frac{[E][S]}{K_S}\right)}{k_3\left(\frac{[E][I]}{\gamma K_i} + [E] + \frac{[E][I]}{K_i} + \frac{[E][S]}{K_S} + \frac{[E][I][S]}{K_S K_i}\right)}$$

The equation above is simplified by eliminating k_3 and [E]

$$\frac{v_0}{V_{max}} = \frac{\frac{[S]}{K_S}}{\left(\frac{[I]}{\gamma K_i} + 1 + \frac{[I]}{K_i} + \frac{[S]}{K_S} + \frac{[I][S]}{K_S K_i}\right)}$$

Numerator and denominator are multiplied by K_s.

$$\frac{v_0}{V_{max}} = \frac{[S]}{\left(\frac{K_S[I]}{\gamma K_i} + K_S + \frac{K_S[I]}{K_i} + [S] + \frac{[I][S]}{K_i}\right)}$$

In the denominator, find a common factor in the terms containing K_s and [S].

$$\frac{v_0}{V_{max}} = \frac{[S]}{K_S\left(1 + \frac{[I]}{\gamma K_i} + \frac{[I]}{K_i}\right) + [S]\left(1 + \frac{[I]}{K_i}\right)}$$

$$v_0 = \frac{V_{max}[S]}{K_S\left(1 + \frac{[I]}{\gamma K_i} + \frac{[I]}{K_i}\right) + [S]\left(1 + \frac{[I]}{K_i}\right)}$$

The equation above is similar to the Michaelis-Menten equation. Hence, it is linearized using the Lineweaver-Burk inversion.

$$\frac{1}{v_0} = \frac{K_S}{V_{max}}\left(1 + \frac{[I]}{\gamma K_i} + \frac{[I]}{K_i}\right)\frac{1}{[S]} + \frac{1}{V_{max}}\left(1 + \frac{[I]}{K_i}\right)$$

Equation 5

Based on the equation 5, by isolating the terms that multiply the slope (K_s/V_{max}) and the intercept ($1/V_{max}$), we can analyze the behavior of these terms as a function of [I].

Slope multiplicative term

$$1 + \frac{[I]}{\gamma K_i} + \frac{[I]}{K_i} = \frac{\gamma K_i + [I] + \gamma[I]}{\gamma K_i} = \frac{\gamma K_i}{\gamma K_i} + \frac{[I](1+\gamma)}{\gamma K_i} = 1 + \frac{(1+\gamma)}{\gamma K_i}[I]$$

Intercept multiplicative term

$$1 + \frac{[I]}{K_i} = \frac{K_i + [I]}{K_i} = \frac{K_i}{K_i} + \frac{[I]}{K_i} = 1 + \frac{1}{K_i}[I]$$

Both terms are linear functions of [I]

Case 3 – General Inhibition Model in 0 < β < 1

Taking the general inhibition model above and assuming 0 < β < 1, the general equation 2 applies.

$$\frac{1}{v_0} = \frac{K_S}{V_{max}}\left(\frac{1+\frac{[I]}{\gamma K_i}+\frac{[I]}{K_i}}{1+\frac{\beta[I]}{K_i}}\right)\frac{1}{[S]} + \frac{1}{V_{max}}\left(\frac{1+\frac{[I]}{K_i}}{1+\frac{\beta[I]}{K_i}}\right)$$

As already demonstrated above, by isolating the term that multiplies the slope (K_s/V_{max}), we can analyze its behavior as a function of [I].

$$multiplicative\ term = \frac{\left(1+\frac{[I]}{\gamma K_i}+\frac{[I]}{K_i}\right)}{\left(1+\frac{\beta[I]}{K_i}\right)}$$

$$if\ [I] \to \infty \ \therefore \ \frac{[I]}{\gamma K_i}+\frac{[I]}{K_i} \gg 1\ and\ \frac{\beta[I]}{K_i} \gg 1$$

$$\frac{\left(1+\frac{[I]}{\gamma K_i}+\frac{[I]}{K_i}\right)}{\left(1+\frac{\beta[I]}{K_i}\right)} = \frac{\left(\frac{[I]}{\gamma K_i}+\frac{[I]}{K_i}\right)}{\left(\frac{\beta[I]}{K_i}\right)} = \frac{\frac{[I]}{K_i}\left(1+\frac{1}{\gamma}\right)}{\frac{[I]}{K_i}(\beta)} = \frac{\gamma+1}{\beta\gamma}$$

Thus, the slope and intercept multiplicative terms have a finite and constant value at infinite [I].

In addition to that, we can simulate the behavior of this term as a function of [I].

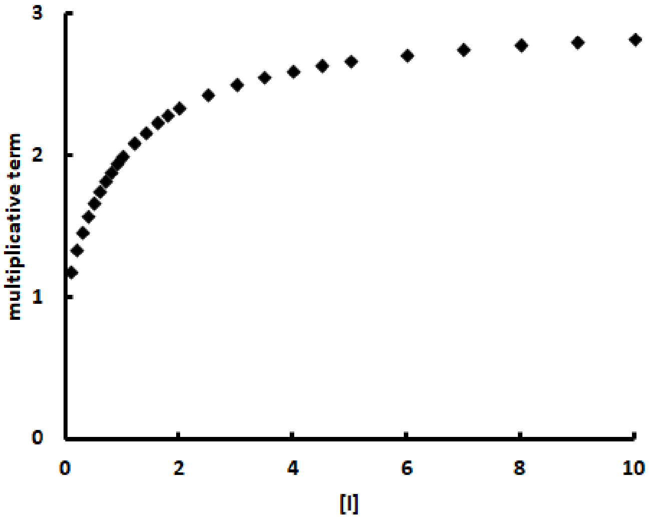

Simulation of the slope multiplicative magnitude as a function of [I]. K_i = 1 and γ = 0.5

Similarly, by isolating the term that multiplies the intercept ($1/V_{max}$), we can analyze its behavior as a function of [I].

$$multiplicative\ term = \frac{\left(1 + \frac{[I]}{K_i}\right)}{\left(1 + \frac{\beta[I]}{K_i}\right)}$$

$$if\ [I] \rightarrow \infty\ \therefore\ \frac{[I]}{K_i} \gg 1\ and\ \frac{\beta[I]}{K_i} \gg 1$$

$$multiplicative\ term = \frac{\left(1 + \frac{[I]}{K_i}\right)}{\left(1 + \frac{\beta[I]}{K_i}\right)} = \frac{\left(\frac{[I]}{K_i}\right)}{\left(\frac{\beta[I]}{K_i}\right)} = \frac{1}{\beta}$$

Thus, the intercept multiplicative term has a finite and constant value at infinite [I].

In addition to that, we can simulate the behavior of this term as a function of [I].

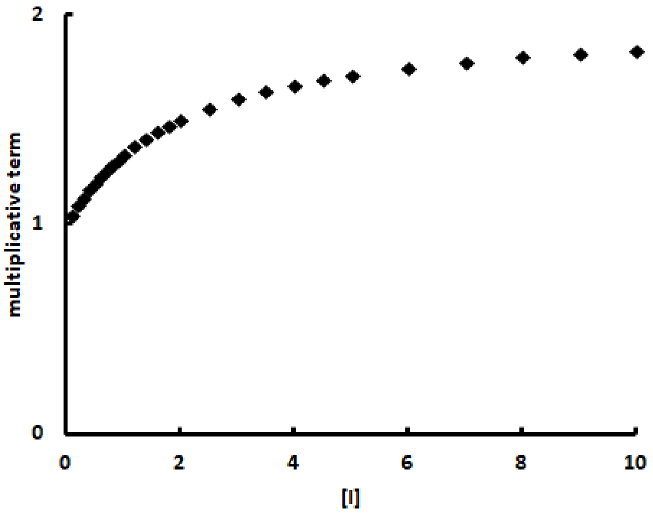

Simulation of the intercept multiplicative magnitude as a function of [I]. K_i = 1 and β = 0.5

Finally, by assuming arbitrary values for the constants in the Lineweaver-Burk equation 2 above, we can trace the lines observed in two different inhibitor concentrations showing that they meet in the second quadrant.

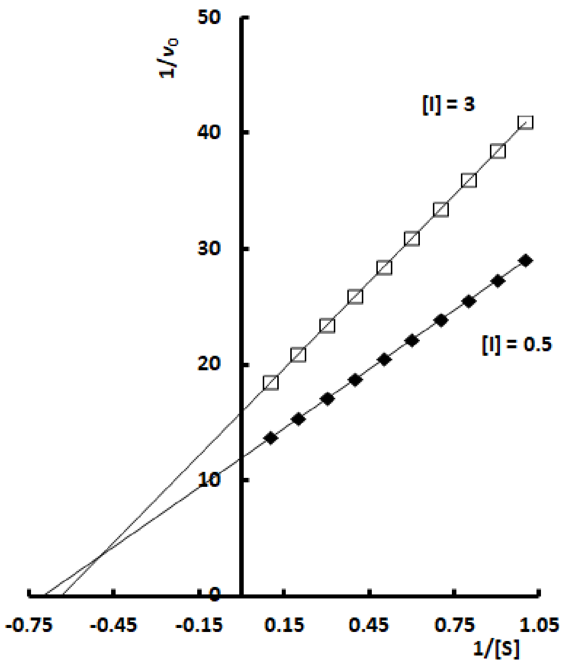

Simulation of the Lineweaver-Burk lines for K_s = 1, V_{max} = 0.1, β = 0, K_i = 1 and γ = 0.5 in two different [I] (0.5 and 3)

Case 4 – General Inhibition Model in β = 1 and γ << 1

Taking the general inhibition model and assuming β = 1, the kinetic equation 2 deduced above is

$$\frac{1}{v_0} = \frac{K_S}{V_{max}}\left(\frac{1+\frac{[I]}{\gamma K_i}+\frac{[I]}{K_i}}{1+\frac{[I]}{K_i}}\right)\frac{1}{[S]}+\frac{1}{V_{max}}$$

Assuming γ << 1 tem factor multiplying the $\frac{K_S}{V_{max}}$ is simplified as follows:

$$\frac{1+\frac{[I]}{\gamma K_i}+\frac{[I]}{K_i}}{1+\frac{[I]}{K_i}}$$

If γ << 1 then $\frac{[I]}{\gamma K_i}$ >> 1 and also $\frac{[I]}{\gamma K_i}$ >> $\frac{[I]}{K_i}$. Thus

$$\frac{\frac{[I]}{\gamma K_i}}{1+\frac{[I]}{K_i}}$$

$$\frac{\frac{[I]}{\gamma K_i}}{\frac{K_i+\ [I]}{K_i}}$$

$$\frac{K_i[I]}{\gamma K_i(K_i+[I])}$$

$$\frac{K_i[I]}{\gamma {K_i}^2+\gamma K_i[I]}$$

$$\frac{[I]}{\gamma K_i+\gamma[I]}$$

$$\frac{1}{\gamma}\left(\frac{[I]}{K_i+[I]}\right)$$

Quando K_i >> [I], então K_i + [I] ≈ K_i

$$\frac{1}{\gamma}\left(\frac{[I]}{K_i+[I]}\right)=\frac{1}{\gamma K_i}[I]$$

In this particular situation, the kinetic equation is written as

$$\frac{1}{v_0} = \frac{K_S}{V_{max}}\left(\frac{1}{\gamma K_i}[I]\right)\frac{1}{[S]}+\frac{1}{V_{max}}$$

Equation 6

Case 5 – General Inhibition Model in β = 0 and γ >> 1

Taking the general inhibition model and assuming β = 0, the kinetic equation 2 deduced above is

$$\frac{1}{v_0} = \frac{K_S}{V_{max}}\left(1 + \frac{[I]}{\gamma K_i} + \frac{[I]}{K_i}\right)\frac{1}{[S]} + \frac{1}{V_{max}}\left(1 + \frac{[I]}{K_i}\right)$$

Assuming γ >> 1 the factor multiplying the $\frac{K_S}{V_{max}}$ is simplified as follows:

$$1 + \frac{[I]}{\gamma K_i} + \frac{[I]}{K_i}$$

If γ >> 1 then $\frac{[I]}{\gamma K_i}$ << 1, then

$$1 + \frac{[I]}{\gamma K_i} + \frac{[I]}{K_i} \approx \; 1 + \frac{[I]}{K_i}$$

So, the kinetic equation is rewritten as:

$$\frac{1}{v_0} = \frac{K_S}{V_{max}}\left(1 + \frac{[I]}{K_i}\right)\frac{1}{[S]} + \frac{1}{V_{max}}\left(1 + \frac{[I]}{K_i}\right)$$

Equation 7

Case 6 – General Inhibition Model in 0 < β < 1 and γ >> 1

Taking the general inhibition model and assuming 0 < β < 1, the kinetic equation 2 deduced above is

$$\frac{1}{v_0} = \frac{K_S}{V_{max}}\left(\frac{1+\frac{[I]}{\gamma K_i}+\frac{[I]}{K_i}}{1+\frac{\beta[I]}{K_i}}\right)\frac{1}{[S]} + \frac{1}{V_{max}}\left(\frac{1+\frac{[I]}{K_i}}{1+\frac{\beta[I]}{K_i}}\right)$$

Assuming γ >> 1 the term multiplying the $\frac{K_S}{V_{max}}$ is simplified as follows:

$$\frac{\left(1+\frac{[I]}{\gamma K_i}+\frac{[I]}{K_i}\right)}{\left(1+\frac{\beta[I]}{K_i}\right)} = \frac{\left(1+\frac{[I]}{K_i}\right)}{\left(1+\frac{\beta[I]}{K_i}\right)}$$

Hence, the slope and intercept are multiplied by the same term. Next we can analyze its behavior as a function of [I].

$$multiplicative\ term = \frac{\left(1+\frac{[I]}{K_i}\right)}{\left(1+\frac{\beta[I]}{K_i}\right)}$$

$$if\ [I] \to \infty \ \therefore\ \frac{[I]}{K_i} \gg 1\ and\ \frac{\beta[I]}{K_i} \gg 1$$

$$multiplicative\ term = \frac{\left(1+\frac{[I]}{K_i}\right)}{\left(1+\frac{\beta[I]}{K_i}\right)} = \frac{\left(\frac{[I]}{K_i}\right)}{\left(\frac{\beta[I]}{K_i}\right)} = \frac{1}{\beta}$$

Thus, the intercept multiplicative term has a finite and constant value at infinite [I].

In addition to that, we can simulate the behavior of this term as a function of [I].

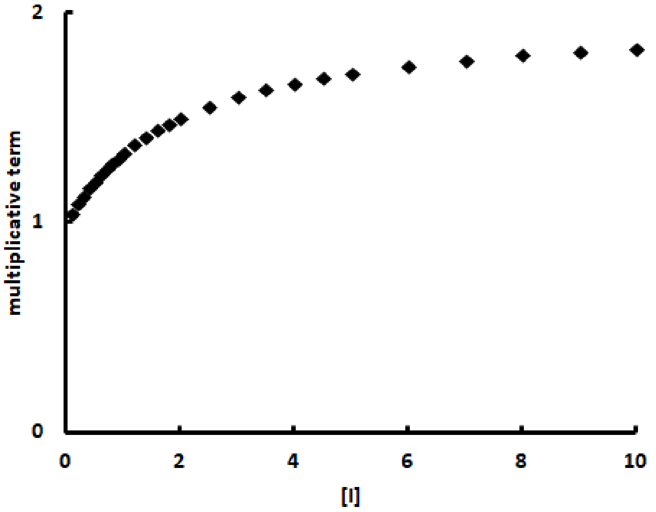

Simulation of the slope and intercept multiplicative terms magnitude as a function of [I]. K_i = 1 and β = 0.5

Correlation between parameters γ and α

Assuming β = 1 the general inhibition model generates the equation 4

$$\frac{1}{v_0} = \frac{K_S}{V_{max}} \left(\frac{1 + \frac{[I]}{\gamma K_i} + \frac{[I]}{K_i}}{1 + \frac{[I]}{K_i}} \right) \frac{1}{[S]} + \frac{1}{V_{max}}$$

By isolating the term that multiplies the slope (K_s/V_{max}) and analyzing its behavior as a function of [I], we conclude that at infinite [I], the term converge to a finite number.

$$multiplicative\ term = \frac{\left(1 + \frac{[I]}{\gamma K_i} + \frac{[I]}{K_i}\right)}{\left(1 + \frac{[I]}{K_i}\right)}$$

$$if\ [I] \to \infty\ \therefore\ \frac{[I]}{\gamma K_i} + \frac{[I]}{K_i} \gg 1\ and\ \frac{[I]}{K_i} \gg 1$$

$$\frac{\left(1 + \frac{[I]}{\gamma K_i} + \frac{[I]}{K_i}\right)}{\left(1 + \frac{[I]}{K_i}\right)} = \frac{\left(\frac{[I]}{\gamma K_i} + \frac{[I]}{K_i}\right)}{\left(\frac{[I]}{K_i}\right)} = \frac{\frac{[I]}{K_i}\left(1 + \frac{1}{\gamma}\right)}{\left(\frac{[I]}{K_i}\right)} = 1 + \frac{1}{\gamma}$$

Similarly, the kinetic equation for a partial competitive mechanism has a term that multiplies the slope (K_s/V_{max}), which also converges to a finite number.

$$if\ [I] \to \infty\ \therefore\ \frac{[I]}{\alpha K_i} \gg 1\ and\ \frac{[I]}{K_i} \gg 1$$

$$\frac{\left(1 + \frac{[I]}{K_i}\right)}{\left(1 + \frac{[I]}{\alpha K_i}\right)} = \frac{\left(\frac{[I]}{K_i}\right)}{\left(\frac{[I]}{\alpha K_i}\right)} = \alpha$$

Hence, both multiplicative terms converge to finite numbers, indicating that

$$\alpha = 1 + \frac{1}{\gamma}$$

Based on this equation, we may simulate the relation between α and γ

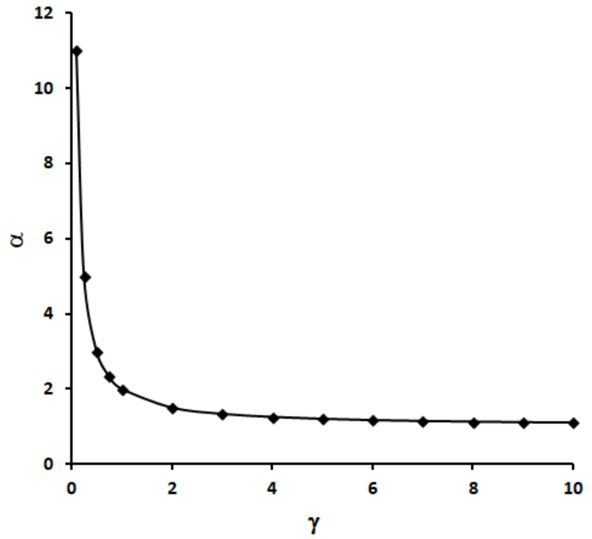

Correlation between parameters γ and α.
