# Supplementary material for: A general model for analysis of linear and hyperbolic enzyme inhibition mechanisms
Source: FEBS Open Bio. 2025 Sep 24;16(2):365–81. doi: 10.1002/2211-5463.70128 (PMC12871559; doi:10.1002/2211-5463.70128)
Supplement: Supplementary file 4 — Fig. S4. Determination of K i using the general inhibition model. [file FEB4-16-365-s002.pdf]

Supplementary Figure 4 – Determination of dissociation constants (K_i and γK_i) and γ using the general inhibition model

Imidazole is a partial competitive inhibitor of Sfβgly (Table 1). This inhibition mechanism is a facet of the general model when $\beta = 1$ (Case 1; Supplementary Figure 3). Assuming this condition, the general equation 1 (main text) is converted in the equation 4:

$$\frac{1}{v_0} = \frac{K_S}{V_{max}}\left(\frac{1 + \frac{[I]}{\gamma K_i} + \frac{[I]}{K_i}}{1 + \frac{[I]}{K_i}}\right)\frac{1}{[S]} + \frac{1}{V_{max}}$$

The slope term is isolated

$$slope = \frac{K_S}{V_{max}}\frac{\left(1 + \frac{[I]}{\gamma K_i} + \frac{[I]}{K_i}\right)}{\left(1 + \frac{[I]}{K_i}\right)}$$

and linearized using the following steps.

$$\Delta slope = \frac{K_S}{V_{max}}\frac{\left(1 + \frac{[I]}{\gamma K_i} + \frac{[I]}{K_i}\right)}{\left(1 + \frac{[I]}{K_i}\right)} - \frac{K_S}{V_{max}}$$

$$\Delta slope = \frac{K_S\left(1 + \frac{[I]}{\gamma K_i} + \frac{[I]}{K_i}\right) - K_S\left(1 + \frac{[I]}{K_i}\right)}{V_{max}\left(1 + \frac{[I]}{K_i}\right)}$$

$$\Delta slope = \frac{K_S\frac{[I]}{\gamma K_i}}{V_{max} + V_{max}\frac{[I]}{K_i}}$$

$$\frac{1}{\Delta slope} = \frac{V_{max} + V_{max}\frac{[I]}{K_i}}{K_S\frac{[I]}{\gamma K_i}}$$

$$\frac{1}{\Delta slope} = \frac{\gamma K_i V_{max} + \gamma V_{max}[I]}{K_S[I]}$$

$$\frac{1}{\Delta slope} = \frac{\gamma K_i V_{max}}{K_S}\frac{1}{[I]} + \frac{\gamma V_{max}}{K_S}$$

So, if 1/Δslope = 0, then 1/[I] = -1/K_i, as shown below.

$$0 = \frac{\gamma K_i V_{max}}{K_S}\frac{1}{[I]} + \frac{\gamma V_{max}}{K_S}$$

$$\frac{1}{[I]} = \frac{\frac{\gamma K_i V_{max}}{K_S}}{-\frac{\gamma V_{max}}{K_S}} = -\frac{1}{K_i}$$

Hence taking the slopes of lines obtained in different [I] of a Lineweaver-Burk plot and then preparing a secondary plot of 1/Δslope *versus* 1/[I], the result is a line that crosses the x-axis at -1/K_i.

On the other side, considering a simple partial competitive mechanism, the plot 1/Δslope *versus* 1/[I] results in a line that crosses the x-axis at -1/αK_i.

Then, both inhibition mechanisms, the general and the simple partial competive, preview that in the plot 1/Δslope *versus* 1/[I] a line will cross the x-axis at the same point. But, that point has different meaning according the mechanism, *i.e,* -1/K_i in the former and -1/αK_i in the second. Thus, it can be stated:

$$\frac{1}{K_i} = \frac{1}{\alpha K_i}$$

Therefore, the αK_i, calculated using the simple partial competitive model (as in Table 1), corresponds to the K_i, if assumed that the general inhibition model (Figure 10) actually describes the enzyme inhibition.

Tris is a linear mixed-type inhibitor of Sfβgly (Table 1). This inhibition mechanism is a facet of the general model when β = 0 (Case 2; Supplementary Figure 3). Assuming this condition, the general equation 1 (main text) is converted in the equation 5:

$$\frac{1}{v_0} = \frac{K_S}{V_{max}}\left(1 + \frac{[I]}{\gamma K_i} + \frac{[I]}{K_i}\right)\frac{1}{[S]} + \frac{1}{V_{max}}\left(1 + \frac{[I]}{K_i}\right)$$

The slope term is isolated

$$slope = \frac{K_S}{V_{max}}\left(1 + \frac{[I]}{\gamma K_i} + \frac{[I]}{K_i}\right)$$

and linearized using the following procedures

$$slope = \frac{K_S}{V_{max}} + \frac{K_S}{V_{max}}\frac{[I]}{\gamma K_i} + \frac{K_S}{V_{max}}\frac{[I]}{K_i} = \frac{K_S}{V_{max}} + \frac{(K_S + \gamma K_S)}{V_{max}\gamma K_i}[I]$$

So, if slope = 0, then then [I] = $\frac{-\gamma K_i}{1+\gamma}$, as shown below

$$0 = \frac{K_S}{V_{max}} + \frac{(K_S + \gamma K_S)}{V_{max}\gamma K_i}[I]$$

$$[I] = \frac{-\frac{K_S}{V_{max}}}{\frac{(K_S + \gamma K_S)}{V_{max}\gamma K_i}}$$

$$[I] = \frac{-V_{max}\gamma K_i K_S}{V_{max}(K_S + \gamma K_S)}$$

$$[I] = \frac{-\gamma K_i}{1 + \gamma}$$

Hence taking the slopes of lines obtained in different [I] of a Lineweaver-Burk plot and then preparing a secondary plot of slope *versus* [I], the result is a line that crosses the x-axis at $\frac{-\gamma K_i}{1+\gamma}$.

On the other side, considering a simple linear mixed mechanism, the plot slope *versus* [I] results in a line that crosses the x-axis at -K_i.

Then both inhibition mechanisms, the general and the simple linear mixed, preview that in the plot slope *versus* [I] a line will cross the x-axis at the same point. But, that point has different meaning according the mechanism, *i.e,* -K_i in the former and $\frac{-\gamma K_i}{1+\gamma}$ in the second. Thus, it can be stated:

$$K_i = \frac{\gamma K_i}{1 + \gamma}$$

Therefore, the K_i, calculated using the simple linear mixed model (as in Table 1), corresponds to the $\frac{\gamma K_i}{1+\gamma}$ if assumed that the general inhibition model (Figure 10) actually describes the enzyme inhibition.. Then, taking the K_i and α presented in Table 1 and also the equation 5 (main text)

$$\alpha = 1 + \frac{1}{\gamma}$$

the γK_i, K_i and γ for the general inhibition model can be calculated.
