# Supplementary material for: A general model for analysis of linear and hyperbolic enzyme inhibition mechanisms
Source: FEBS Open Bio. 2025 Sep 24;16(2):365–81. doi: 10.1002/2211-5463.70128 (PMC12871559; doi:10.1002/2211-5463.70128)
Supplement: Supplementary file 5 — Data S1. Data used in the determination of the imidazole and Tris inhibition mechanisms of the mutant Sfβgly. [file FEB4-16-365-s005.pdf]

DATA SET

Data used in the determination of the Imidazole inhibition mechanism of the mutant Sfβgly S247Y. This data is presented in the Figure 2, in which different Imidazole concentrations are shown as ●, 0; ■, 20 mM; ◆, 40 mM; **x**, 80 mM; ○, 120 mM. Initial rates (v_0) of substrate hydrolysis (μM.min^{-1}) are the average of three separate assays. Yellow cells mark rates discarded in the final analysis.

	0	20	40	80	120
1/[S]	1/v0	1/v0	1/v0	1/v0	1/v0
4.00	1.289617	1.616438	1.6	2.458333	1.416
2.50	1.099379	0.817552	1.356322	2.107143	2.466899
1.67	0.946524	1.204082	1.299083	1.815385	2.226415
1.25	0.866585	0.932806	1.296703	1.646512	2
1.00	0.84994	1.051225	1.216495	1.535792	1.955801
0.83	0.838863	0.95935	1.134615	1.481172	1.853403
0.63	0.827103	0.988827	1.067873	1.369439	1.77
0.50	0.830012	0.987448	1.052006	1.353728	1.765586
0.40	0.800905	0.949062	1.014327	1.303867	1.677725
0.33	0.812859	0.936508	0.903061	1.253097	1.646512
0.25	0.759657	0.892812	0.923077	1.204082	1.573333

Data used in the determination of the Imidazole inhibition mechanism of the mutant Sfβgly N249Q. This data is presented in the Figure 3, in which different Imidazole concentrations are shown as ●, 0; ■, 20 mM; ◆, 40 mM; **x**, 80 mM; ○, 120 mM. Initial rates (v_0) of substrate hydrolysis (μM.min^{-1}) are the average of three separate assays. Yellow cells mark rates discarded in the final analysis.

	0	20	40	80	120
1/[S]	1/v0	1/v0	1/v0	1/v0	1/v0
4.00	0.07615	0.1416	0.14538	0.148272	0.165808
2.50	0.056902	0.089394	0.103888	0.141317	0.152013
1.67	0.045847	0.065193	0.085585	0.100675	0.127224
1.25	0.041061	0.057491	0.0705	0.075702	0.097588
1.00	0.037615	0.050635	0.061299	0.073444	0.085122
0.83	0.03489	0.044002	0.052078	0.061565	0.072597
0.63	0.032888	0.03977	0.046679	0.05463	0.061713
0.50	0.030951	0.036736	0.041672	0.048777	0.05443
0.40	0.031036	0.034248	0.03752	0.041616	0.047903
0.33	0.030273	0.033558	0.035731	0.039503	0.044215
0.25	0.029714	0.031446	0.033503	0.036303	0.039481
0.17	0.02928	0.030498	0.031678	0.033119	0.034199
0.10	0.028766	0.029658	0.0312	0.030025	0.031097

Data used in the determination of the Imidazole inhibition mechanism of the mutant Sfβgly N249A. This data is presented in the Figure 4, in which different Imidazole concentrations are shown as ●, 0; ■, 20 mM; ◆, 40 mM; **x**, 80 mM; ○, 120 mM. Initial rates (v_0) of substrate hydrolysis (μM.min^{-1}) are the average of three separate assays. Yellow cells mark rates discarded in the final analysis. Orange cells indicate failed assays.

	0	20	40	80	120
1/[S]	1/v0	1/v0	1/v0	1/v0	1/v0
4.00	0.219365	0.356226	0.310526	0.634978	0.840356
2.50	0.160818	0.342857	0.375099	0.666353	0.636404
1.67	0.13246	0.287513	0.332005	0.53434	
1.25	0.10981	0.222817	0.311209	0.43105	
1.00	0.098917	0.18994	0.253536	0.352677	
0.83	0.09168	0.166883	0.215525	0.297167	
0.63	0.090277	0.154501	0.172788	0.265915	0.243508
0.50	0.083936	0.131415	0.156119	0.238786	0.273359
0.40	0.075179	0.125421	0.145754	0.218856	0.237385
0.33	0.077419		0.133837	0.182828	0.216679
0.25	0.066448	0.094558	0.131599	0.180728	0.185219
0.17	0.060877	0.089819	0.097622	0.158478	0.13999
0.10	0.057235	0.078974	0.093744	0.102201	0.124374

Data used in the determination of the Imidazole inhibition mechanism of the mutant Sfβgly F251A. This data is presented in the Figure 5, in which different Imidazole concentrations are shown as ●, 0; ■, 20 mM; ◆, 40 mM; **x**, 80 mM; ○, 120 mM. Initial rates (v_0) of substrate hydrolysis (μM.min^{-1}) are the average of three separate assays. Yellow cells mark rates discarded in the final analysis.

	**0**	**20**	**40**	**80**	**120**
**1/[S]**	**1/v0**	**1/v0**	**1/v0**	**1/v0**	**1/v0**
4.00	0.204499	0.3210884	0.3955307	0.433691	0.54357
2.50	0.173913	0.2659155	0.3247706	0.414035	0.408658
1.67	0.156495	0.222292	0.2699714	0.234631	0.265169
1.25	0.143885	0.1984583	0.2160183	0.297167	0.304844
1.00	0.140845	0.1835386	0.2215962	0.253083	0.266416
0.83	0.118133	0.1713249	0.1984583	0.227105	0.236394
0.63	0.124844	0.1568975	0.1799238	0.207018	0.207625
0.50	0.117028	0.1498413	0.169378	0.163889	0.199016
0.40	0.113122	0.1338374	0.1611838	0.174277	0.180038
0.33	0.107701	0.1301471	0.1492884	0.169886	0.182828
0.25	0.100959	0.1235063	0.1366136	0.14267	0.167773
0.17	0.088067	0.1030943	0.1451563	0.139748	0.1475
0.10	0.078958	0.0889727	0.1003188	0.113598	0.120306

Data used in the determination of the Tris inhibition mechanism of the mutant Sfβgly S247Y. This data is presented in the Figure 6, in which different Imidazole concentrations are shown as ●, 0; ■, 20 mM; ◆, 40 mM; **x**, 80 mM; ○, 120 mM. Initial rates (v_0) of substrate hydrolysis (μM.min^{-1}) are the average of three separate assays. Yellow cells mark rates discarded in the final analysis.

	0	20	40	80	120
1/[S]	1/v0	1/v0	1/v0	1/v0	1/v0
12.50	2.171779	2.449827	2.733591	3.051724	3.54
8.33	1.735294	2	2.283871	2.254777	1.583893
6.25	1.545852	1.706024	1.945055	2.254777	2.692015
5.00	1.377432	1.556044	1.783375	1.955801	2.424658
4.00	1.253097	1.351145	1.569845	1.765586	2.158537
2.50	1.055142	0.923077	1.289617	1.410359	1.335849
1.67	0.941489	1.17608	1.114961	1.195946	1.39645
1.25	0.863415	0.946524	1.020173	1.069486	1.212329
1.00	0.835891	0.920078	0.950336	0.952894	1.158756
0.83	0.803632	0.874074	0.900763	0.95935	1.047337
0.63	0.851986	0.853012	0.883895	0.927916	1.011429
0.50	0.669187	0.838863	0.869779	0.664165	1.033577
0.40	0.772926	0.806378	0.842857	0.671727	0.915912
0.33	0.73904	0.775465	0.820394	0.811927	0.871921
0.25	0.728395	0.691406	0.781457	0.75641	0.827103
0.17	0.695481	0.731405	0.741361	0.724667	0.778022
0.10	0.672365	0.706587	0.698225	0.622691	0.741361

Data used in the determination of the Tris inhibition mechanism of the mutant Sfβgly N249Q. This data is presented in the Figure 7, in which different Imidazole concentrations are shown as ●, 0; ■, 20 mM; ◆, 40 mM; **x**, 80 mM; ○, 120 mM. Initial rates (v_0) of substrate hydrolysis (μM.min^{-1}) are the average of three separate assays. Yellow cells mark rates discarded in the final analysis.

	0	20	40	80	120
**1/[S]**	**1/v0**	**1/v0**	**1/v0**	**1/v0**	**1/v0**
4.00	0.098814	0.13896	0.153579	0.247769	0.308161
2.50	0.066557	0.098095	0.118246	0.180267	0.232131
1.67	0.052522	0.076129	0.087597	0.130627	0.163134
1.25	0.042294	0.059334	0.070116	0.104772	0.130748
1.00	0.035948	0.049381	0.05981	0.084968	0.109853
0.83	0.034993	0.045153	0.054482	0.075399	0.096033
0.63	0.033373	0.038901	0.045833	0.060629	0.080045
0.50	0.031148	0.036229	0.041488	0.055022	0.069857
0.40	0.030753	0.035281	0.040319	0.052097	0.062406
0.33	0.030153	0.033236	0.037352	0.046725	0.056168
0.25	0.02886	0.031231	0.034629	0.042112	0.049047
0.17	0.029007	0.030282	0.034034	0.037993	0.042351
0.10	0.027719	0.029559	0.03026	0.035071	0.038689

Data used in the determination of the Tris inhibition mechanism of the mutant Sfβgly N249A. This data is presented in the Figure 8, in which different Imidazole concentrations are shown as ●, 0; ■, 20 mM; ◆, 40 mM; **x**, 80 mM; ○, 120 mM. Initial rates (v_0) of substrate hydrolysis (μM.min^{-1}) are the average of three separate assays. Yellow cells mark rates discarded in the final analysis.

	0	20	40	80	120
1/[S]	1/v0	1/v0	1/v0	1/v0	1/v0
4.00	0.167376	0.335943	0.569819	0.901911	1.12381
2.50	0.136023	0.301919	0.417699	0.331228	0.622418
1.67	0.111981	0.243928	0.3776	0.583918	0.486598
1.25	0.094779	0.186931	0.276833	0.445984	0.674286
1.00	0.081356	0.156941	0.224228	0.366365	0.55748
0.83	0.075742	0.143175	0.200851	0.311551	0.440435
0.63	0.096688	0.173317	0.220389	0.339568	0.501239
0.50	0.085071	0.147731	0.191222	0.253991	0.355332
0.40	0.077931	0.118593	0.167673	0.203011	0.356226
0.33	0.068044	0.105045	0.157948	0.224228	0.234243
0.25	0.060642	0.086368	0.123452	0.186807	0.253763
0.17	0.053789	0.07617	0.103207	0.140407	0.161092
0.10	0.050168	0.066682	0.08875	0.111672	0.126485

Data used in the determination of the Tris inhibition mechanism of the mutant Sfβgly F251A. This data is presented in the Figure 9, in which different Imidazole concentrations are shown as ●, 0; ■, 20 mM; ◆, 40 mM; **x**, 80 mM; ○, 120 mM. Initial rates (v_0) of substrate hydrolysis (μM.min^{-1}) are the average of three separate assays. Yellow cells mark rates discarded in the final analysis.

	0	20	40	80	120
1/[S]	1/v0	1/v0	1/v0	1/v0	1/v0
4.00	0.222467	0.515847	0.70099	0.997183	0.899048
2.50	0.175465	0.32	0.556385	0.816138	1.085057
1.67	0.153371	0.317845	0.469652	0.622418	0.444584
1.25	0.139233	0.230807	0.361224	0.545665	0.708
1.00	0.135115	0.230431	0.333962	0.472	0.576782
0.83	0.124923	0.177666	0.290462	0.423318	0.560792
0.63	0.119898	0.162852	0.247552	0.356675	0.464262
0.50	0.10707	0.160909	0.223874	0.320725	0.311551
0.40	0.108547	0.135762	0.205366	0.281231	0.406897
0.33	0.108589	0.144896	0.186807	0.231373	0.354
0.25	0.100855	0.135762	0.163794	0.223874	0.309847
0.17	0.086898	0.10366	0.137276	0.189052	0.243508
0.10	0.076748	0.10332	0.111848	0.154585	0.196394
